# Meeting Report: The Allied Genetics Conference 2016

**DOI:** 10.1534/g3.116.036848

**Published:** 2016-12-06

**Authors:** Organizers of The Allied Genetics Conference 2016

**Affiliations:** Genetics Society of America, Bethesda, Maryland, 20814

**Keywords:** Caenorhabditis, ciliates, Drosophila, mouse, yeast, zebrafish, population genetics, evolutionary genetics, quantitative genetics

The mission of the Genetics Society of America (GSA) is to deepen our understanding of the living world by advancing the field of genetics. A cornerstone of this mission is promoting interaction among geneticists. To foster such connections and collaborations across the field, the GSA brought together seven research communities for The Allied Genetics Conference (TAGC), July 13–17, 2016 in Orlando, Florida (http://genetics2016.org). The individual meetings hosted at TAGC focused on the genetics of *Caenorhabditis elegans*, ciliates, *Drosophila*, mouse, yeast, and zebrafish, as well as population, evolutionary, and quantitative genetics. In addition to community-specific programming, the groups came together each day for an outstanding series of keynote presentations held in joint plenary sessions. Members of all communities also took part in shared education and professional development events.

This report includes highlights contributed by the organizing committees of each community meeting, an update on model organism database news from TAGC, summaries of talks by Jef Boeke, Francis Collins, Jennifer Doudna, Anna-Katerina Hadjantonakis, David Kingsley, Laura Landweber/Richard Miller, Michael Miller, Molly Przeworski, Pamela Ronald, Amita Sehgal, and Leonard Zon, followed by a list of Honors and Awards conferred at the meeting.

## C. elegans Development, Cell Biology, and Gene Expression Meeting

The *C. elegans* Development, Cell Biology, and Gene Expression Meeting at TAGC featured a number of presentations that represented significant scientific and technical advances. Like its fellow model systems, *C. elegans* has experienced a revolution in genetics due to the development of powerful genome engineering techniques. A Clustered Regularly Interspaced Short Palindromic Repeats (CRISPR) workshop organized by Mike Boxem (Utrecht University) featured presentations by Alex Paix (Johns Hopkins University), who reported on the use of a highly efficient and versatile recombineering approach for making genome modifications, and a talk by Daniel Dickinson (The University of North Carolina) who described improvements to his streamlined method of using a self-excising drug selection cassette for deriving a transcriptional reporter, a knockout allele, and a translational fusion of any gene, all from a single initial CRISPR-mediated insertion. Meanwhile, Matthew Schwartz (HHMI/University of Utah) addressed one of the more time-consuming aspects of genome engineering: construction of repair templates. Using a Golden Gate Cloning strategy, he developed a system for rapidly building complex repair plasmids from multiple interchangeable subunits. One particularly important application of this approach was the use of the FLP recombinase system for tissue-specific expression of transgenes. Finally, Adam Norris (Harvard University) reported on a dual screenable/selectable marker strategy for the highly efficient production of gene knockouts up to an amazing 11.5 kb.

In addition to refinements in genome engineering technology, the meeting ushered in several other technical achievements of note. Worms have lacked several powerful inducible gene expression technologies available in other model systems. Jonathan Liu (California Institute of Technology) developed a Gal4/UAS system derived from the yeast *Saccharomyces kudriavzevii*, and was able to demonstrate inducible tissue-specific expression of a transgene. Similarly, Peter Askjaer (Pablo de Olavide University) optimized FLP recombinase for use in *C. elegans*, and was able to attain spatiotemporal control of gene expression. Using either of these systems, the ability to control gene expression in a cell type- and developmental stage-dependent manner will undoubtedly open up new avenues of inquiry. Daniel Dickinson (The University of North Carolina) presented a single-molecule approach for quantifying protein–protein interactions in individual, staged *C. elegans* embryos, which was used to identify PAR protein complexes that are dynamically regulated during cell polarization.

Among other highlights of the meeting were the three keynote addresses. Michael Miller (University of Alabama at Birmingham) described a role for chemoreceptors of gustatory sensory cilia in modulating sperm behavior. In response to certain pathogenic bacteria, these chemoreceptors elevate oxidative metabolism gene expression during spermatogenesis, resulting in the production of sperm with an enhanced ability to locate a mature oocyte (for more details see talk summary below). Oliver Hobert (Columbia University) presented a tour-de-force effort to provide a comprehensive neurotransmitter map of the *C. elegans* nervous system. His group has assigned neurotransmitter identity to an astounding 90% of the nervous system, and for almost three quarters of the neurons, they have determined how neurotransmitter identity is genetically specified. Barbara Conradt (Ludwig Maximilians University of Munich) described a multi-layered model of a cell death decision. The mother of the dying cell, in coordination with the engulfment pathways, activates the cell death machinery and predetermines which cell will undergo apoptosis after division. A microRNA-based mechanism prevents the mother cell from being killed by the activated cell death machinery.

As the meeting showed, *C. elegans* continues to be a powerful genetic model system, and with an impressive set of new genetic tools, it is likely to remain so for the foreseeable future. Our community will meet again next year at the 21st International *C. elegans* Conference, June 21–25, 2017 at the University of California, Los Angeles.

## Ciliate Molecular Biology CONFERENCE

The Ciliate Molecular Biology (CMB) community has held biannual meetings since the early 1980s. The 2016 gathering, the first organized through GSA, met expectations for disseminating research findings and provided ample opportunity to discuss community-led initiatives and identify infrastructure needs. Importantly, the TAGC venue fostered networking with colleagues working in other model systems. The panel discussion “Speaking Up for Genetics and Model Organism Research” was particularly useful for articulating challenges that each research community faces, with common themes emerging. The “Why Ciliates” video provides a compelling approach to advocating for research to the general public, legislators, and administrators. The video can be viewed at https://vimeo.com/174609655.

The meeting organizers invited three researchers from outside the CMB community to give plenary talks: Susan Dutcher (Washington University at St. Louis; cilia biogenesis), David Bilder (University of California, Berkeley; cell morphogenesis) and Ellen Pritham (University of Utah; mobile DNA). Susan Dutcher described her work in the model system *Chlamydomonas reinhardtii* on cytoplasmic assembly of dynein arms and formation of the transition zone at the base of the cilium. In a complementary talk, Anne-Marie Tassin (CNRS, Gif sur Yvette) described how Cep290 cooperates with two transition zone modules, MKS2 and NPHP, to generate the *Paramecium tetraurelia* ciliary gate. Chad Pearson (University of Colorado Health Science Center) described how Poc1p and Fob1p work together to stabilize radially symmetric triplet microtubules in *Tetrahymena thermophila*. Yu-Yang Jiang (University of Georgia) described a forward genetic approach to isolating suppressors of cilia length misregulation. This effort resulted in the cloning of the *Tetrahymena* ortholog of the *Chlamydomonas* long flagella four gene.

Ciliated protozoa are a rich resource for studying genome rearrangements, as tens of thousands of breakage and rejoining reactions occur to transform a transcriptionally silent “germline” micronucleus into a transcriptionally active “somatic” macronucleus. Ellen Pritham discussed how different classes of mobile genetic elements from nonciliate eukaryotic species were found to carry host cellular genes. In these species, mobile DNA proliferation has created large gene families. Like bacterial contemporaries, eukaryotic mobile DNA serves as a vector for horizontal gene movement between species. Mireille Betermier (CNRS, University of Paris-Sud) provided mechanistic insights into a “domesticated” PiggyBac transposase in *Paramecium* that works in concert with the double-strand break repair complex KU70/80 to break and rejoin DNA during programmed DNA elimination. Aditi Singh (University of Bern) described the contribution of the chromatin remodeler SDGP (ISWI gene family) to the excision of short internally eliminated sequences (IESs) and larger TEs in the developing *Paramecium* macronucleus. Eric Meyer (Institut de Biologie de l’ENS, IBENS) discussed the role of scnRNAs in the removal of transposon-derived *Paramecium* sequences, and Andrea Frapporti (Institut Jaques Monod) provided evidence for transcriptional repression of TEs by the chromatin remodeler enhancer of zeste-like protein 1 (Ezl1). Doug Chalker (Washington University, St. Louis) talked about the role of the G-quadraplex binding protein Lia3, and the related Ltl1, in the removal of different classes of IESs in *Tetrahymena*.

Whole genome sequencing (WGS) is becoming the preferred method to map mutations generated in forward genetic studies. Three WGS analyses described at the meeting identified genes involved in cell division (CDA1; Y. Jiang, University of Georgia), protein trafficking (VPS8; D. Sparvoli, University of Chicago), and cilia biogenesis (LF4; Y. Jiang, University of Georgia). WGS was also used to examine the evolutionary history (expansion and contraction) of *Paramecium* IESs (D. Sellis, Université Lyon 1), and study potential driving forces for the maintenance and loss of duplicated genes. RNA-seq also played a prominent role in many of the meeting talks. This included transcriptome analysis of the unconventional *T. thermophila* cell cycle, in which the micronucleus and macronucleus replicate their DNA and partition their chromosomes at different cell cycle stages (L. Zhang, Texas A&M University). Other RNA-seq projects studied cold adaptation in the Antarctic ciliate *Euplote focardii* (C. Miceli, University of Camerino), sexual/asexual dimorphism in *Tetrahymena* (W. Miao, Institute of Hydrobiology, Chinese Academy of Sciences), and transcription in *P. aurelia* complex species that have diverged following an ancestral whole genome duplication (J. Gout, Indiana University). *Tetrahymena* Genome Database curator Naomi Stover (Bradley University) discussed a recent upgrade to community-based annotation by addition of Web Apollo. Data analysis can now be performed using the software TetraMine. This InterMine will enhance comparative genomics and transcriptomics analyses across species by providing a universal platform for data processing and analysis. On the more translational front, Janna Bednenko (Tetragenetics Inc.) reported on major improvements in protein overproduction in *Tetrahymena*, including the expression and purification of mammalian ion channels. Finally, while conventional reverse genetic approaches can be used to generate targeted gene disruptions and replacements by homologous recombination in *Tetrahymena*, Cas9/CRISPR technology could speed up the process, as well as provide opportunities for reverse genetics in less amenable ciliates. Rachel Howard-Till (University of Vienna) described the use of Cas9/CRISPR to study the role of a condensin involved in macronuclear development and DNA deletion.

The next CMB Conference will be held in the summer of 2018 (date and US site to be determined). We are pleased that the meeting will be sponsored by the GSA and look forward to strengthening our affiliation. The 2018 meeting organizers are Chad Pearson (University of Colorado Health Science Center), Naomi Stover (Bradley University), and Martin Simon (Saarland University, Germany).

## 57th Annual Drosophila Research Conference

This year’s meeting was chaired by David Bilder, Nancy Bonini, Ross Cagan, and Susan Celniker. The opening session featured a tribute to the *Drosophila* colleagues who passed away this year, with a special tribute by Thom Kaufman to William Gelbart, an influential contributor to the fly research community. The tribute was followed by presentation of the *Drosophila* Image Award, the Larry Sandler Award, and the “Discovery of the Homeobox” panel discussion. The Larry Sandler Award Memorial Lecture was given by Alejandra Figueroa-Clarevega (University of California, Berkeley), who spoke on tumor–host interactions, identifying a tumor-secreted factor that interrupts insulin signaling to induce host tissue wasting. The *Hox* panel consisted of three of the key researchers involved in discovering the homeobox in 1983: Matthew Scott (Carnegie Institution for Science), Michael Levine (Princeton University), and William McGinnis (University of California, San Diego). Cassandra Extavour served as an exciting moderator of the panel, having been trained by Antonio Garcia-Bellido, a postdoc of the father of developmental genetics, Edward Lewis. They reflected on the historical context of the independent discovery of the homeobox by two different teams, and the profound importance of the discovery in understanding animal development. New facets in this understanding were revealed in a plenary presentation given later in the week by Ingrid Lohmann (Ruprecht-Karls University). Lohmann spoke on the mechanisms that allow HOX factors to acquire exquisite spatial and temporal specificity, triggering diverse developmental programs with resolutions that can reach the single-cell level. Using larval feeding as a model system, Lohmann’s group proposes that HOX proteins may guide the recognition of interacting synaptic partners to regulate regional motor outputs.

Among the many other highlights of the meeting, Amita Seghal (University of Pennsylvania) gave a keynote presentation on the molecular mechanisms of sleep (see summary in section below). Duojia Pan (Johns Hopkins University) spoke on Hippo signaling and expanding the physiological function of the pathway to many physiological processes beyond growth control. His group has linked the Hippo pathway to Toll signaling in the control of innate immunity. Alain Vincent (CNRS/University Toulouse 3) described a transcription factor code dynamically controlling muscle identity, revealing a cascade of regulatory interactions across position and developmental time. Jian Zhou (Princeton University) reported on efforts to systematically predict gene expression patterns in embryonic development. Zhou and colleagues used a computational approach to integrating cell lineage, genome-wide expression, and chromatin status data, providing comprehensive predictions for tissue-specific expression and a tool for exploratory analysis. Many new avenues of research were also explored in workshops, including well-attended sessions on the *Drosophila* Microbiota and the modMetabolome Model Organism Metabolomics Consortium.

We look forward to meeting again next year at the 58th Annual *Drosophila* Research Conference, to be held March 29–April 2, 2017 in San Diego, CA.

## Mouse Genetics 2016

Mouse Genetics 2016 combined the 30th meeting of the International Mammalian Genome Society (IMGS) with the annual Mouse Molecular Genetics Meeting to provide a stimulating environment for sharing recent discoveries and developing new ideas and collaborations. Two named keynote lectures anchored the meeting: Joe Nadeau (Pacific Northwest Diabetes Research Institute) gave a lecture in honor of Verne Chapman, a founding member of the IMGS, in the “Translational and Systems Genetics” session. Nadeau told an intriguing story of epistasis, transgenerational epigenetics, and fertilization bias. Anna-Katerina Hadjantonakis (Memorial Sloan Kettering Cancer Center) gave a lecture in honor of Rosa Beddington, a leading mouse experimental embryologist. Using state-of-the-art imaging techniques, Hadjantonakis’ work showed that embryonic cells do not behave as classical experiments would suggest (for more details see summary below). In the “Stem Cells” session, Josh Brickman (Danish Stem Cell Institute) showed how embryonic stem cells can stall for time while making key decisions.

The International Mouse Phenotyping Consortium provided updates in the “International Resources” session, revealing a high-throughput imaging platform for mouse embryos that facilitates detection of anomalies by nonspecialist groups. The “Technological Innovations” session continued this theme by promoting new embryo imaging techniques and CRISPR/Cas9 genome editing. In the “Development” session, Takashi Hiiragi (European Molecular Biology Laboratory, EMBL) showed that mechanical forces regulate asymmetric cell divisions (Maître *et al.* 2016). “Comparative Genomics, Computational Methods, and Evolution” featured speaker Hopi Hoekstra (Harvard University), who described genes that regulate parenting behavior (“good dad, bad dad”) in deer mouse fathers. Freda Miller (SickKids Hospital Research Institute) showed how basic research in mouse models has led to clinical trials for brain injury cases in “Human Disease Models.” In “Epigenetics,” Jeannie Lee (Harvard University) demonstrated that the mouse X chromosome can still be partially dosage compensated (inactivated) when the noncoding RNA XIST was eliminated in early mouse development.

Tyler Jacks (Massachusetts Institute of Technology) described how using *in situ* genome editing to engineer mouse cancer models has led to immune-targeted therapy in “Cancer and Immunology.”

Keeping with the multi-organism theme of TAGC, workshops focused on cross-organism approaches and resources, such as genome editing, model organism databases (MODs), cell competition, and genetic reference populations. Mouse Genetics 2016 was preceded by a satellite symposium featuring trainee talks, five of which were selected for podium presentations in the main meeting. IMGS and GSA awards were given for top trainee talks and poster presentations. Jacob Moskowitz (University of Missouri) won the coveted “Verne Chapman Young Investigator” prize, and Krista Geister (Seattle Children’s Research Institute) received an award in honor of Mary Lyon for the top talk by a woman postdoctoral fellow.

Mouse Genetics 2016 attendees left Orlando inspired and looking forward to the next meeting, an EMBL Conference: “Mammalian Genetics and Genomics: From Molecular Mechanisms to Translational Applications,” which will be held at the EMBL Advanced Training Centre, Heidelberg, Germany, October 24–27, 2017.

## Population, Evolutionary, and Quantitative Genetics Conference

The first Population, Evolutionary, and Quantitative (PEQ) Genetics Conference was a resounding success, serving as a hub that drew in many attendees from the six model organism-focused meetings, alongside those focused on nonmodel species. Many attendees appreciated having the program organized by genetic theme, and were excited to be introduced to new systems and unusual organisms. The meeting was launched with a keynote presentation by Patricia Wittkopp (University of Michigan) on using the yeast *S. cerevisiae* to investigate how mutation and selection affect the evolution of gene expression.

A symposium the following day featured candidates for the James F. Crow Early Career Researcher Award, which honors the legacy of population genetics pioneer Jim Crow on the centennial of his birth. In a funny and moving tribute to this legacy, Daniel Hartl (Harvard University) talked of the valuable lessons he had learned from Crow, his PhD mentor, including treasuring your students. The symposium continued with six talented students and recent PhDs presenting on a diverse range of topics. Matthew Ackerman (Indiana University) spoke on a method for estimating pairwise relatedness coefficients from population genomic data. Emily Behrman (University of Pennsylvania) revealed striking seasonal oscillations in selection and adaptation in *Drosophila melanogaster* populations. Heath Blackmon (University of Minnesota) presented results from more than 1000 beetle species to explore the role of Y-aneuploidy in the evolution of sex chromosomes and genome architecture. Gili Greenbaum (Ben Gurion University) described a network theory-based approach to population structure analysis that does not require unrealistic assumptions. Sarah Sander (Cornell University), later announced as winner of the Crow Award, spoke on using fireflies as a model system for understanding animal signal and receptor evolution. Sandeep Venkataram (Stanford University) explored fitness pleiotropy and adaptive mutations in yeast using experimental evolution and high resolution lineage tracing.

In a keynote session focused on complex traits, Dirk-Jan de Koning (Swedish University of Agricultural Sciences) highlighted an example of systems genetics in an applied context, using QTL mapping and GWAS to improve bone strength in egg-laying hens. Among the many other highlights of the meeting, Philipp Messer (Cornell University) described a population genetic model for CRISPR/Cas9-mediated gene drives. The results could be used to design more successful drive strategies or to incorporate mechanisms to limit the spread of a drive. Daniel Skelly (Duke University) presented a creative approach to mapping complex traits in yeast, combining a genetically diverse panel of cross-segregants and a method to deconvolve haplotype blocks from pooled libraries of unbarcoded strains. Beth Dumont (North Carolina State University) creatively used data from the Collaborative Cross to show that a locus on the X chromosome influences the genome-wide rate of meiotic recombination in female mice. Alexander Platt (Temple University) proposed a novel method for estimating the age of singleton or very rare alleles in a population using molecular marker information, drawing information from variants linked to the allele. Yun Ding (HHMI) described how quantifying variation in *Drosophila* courtship songs allowed genetic dissection of a behavioral difference. Richard Durbin (Wellcome Trust Sanger Institute) spoke on WGS of the cichlid fish species famous for their dramatic evolutionary radiation. Annalise Paaby (Georgia Institute of Technology) presented evidence that cryptic genetic variation is ubiquitous in wild *C. elegans*, in the form of alleles that have no effect unless the function of other genes is perturbed. The final keynote was given by John Willis (Duke University), on evidence for parallel genetic mechanisms in repeated adaptation of *Mimulus guttatus* to patches of serpentine soils and toxic mine tailings.

The PEQ program ended with a stimulating session on epistasis, including a talk from Christian Landry (Université Laval), who explored the fate of duplicated yeast genes by examining compensatory changes in protein interaction networks. Gregory Lang (Lehigh University) investigated large-scale patterns of epistasis in yeast by measuring evolutionary trajectories in large pools of recombinant progeny containing random combinations of evolved mutations. David Rand (Brown University) spoke on epistasis between mitochondrial and nuclear genomes in *Drosophila*, finding that interactions were highly dependent on environmental background.

The TAGC attendee survey revealed strong interest from the community in holding another PEQ meeting, although the details are still to be decided.

## Yeast Genetics Meeting

The Yeast Genetics Meeting was marked by 10 scientific sessions and two workshop sessions covering diverse aspects of transcriptional regulation, development, cell division, the stress response, intracellular trafficking, evolution, and new technologies. The conference opened with a session on genome dynamics punctuated by the Winge-Lindegren address of Rodney Rothstein (Columbia University Medical Center), which beautifully built on construction of the widely-used W303 strain to reveal the choreography of the DNA damage response and the mechanisms of homologous recombination. Day 2 saw an exciting session on post-transcriptional gene regulation, including Daniel Jarosz (Stanford University) describing the identification of ∼50 new yeast prions based on a transient overexpression of intrinsically disordered proteins. The “Epigenetics and Transcriptional Regulation” session featured new evidence presented by Ryan Janke (University of California, Berkeley) that the oncometabolite 2-hydroxyglutarate promotes conserved disruption of histone demethylation. The same work identified new metabolites with the potential to cause epigenetic instability. Thursday culminated with a session on human disease modeling, marked by the Lee Hartwell lecture delivered by Susan Gasser (Friedrich Miescher Institute for Biomedical Research) on the *in vivo* dynamics of chromatin, including striking new results on large-scale histone depletion after DNA damage. Day 3 began with “Division and Development,” including Michael McMurray (University of Colorado Anschutz Medical Campus), who described a first detailed analysis of the effects of the ascus on germination and mating behavior between sisters. Jessica Lao (University of California, San Francisco) described the first evidence of direct regulation of RNA degradation machinery by the DNA damage checkpoint. In the same session, James Broach (Penn State College of Medicine) received a lifetime achievement award and reviewed his long career using yeast to understand the biology of nutrient depletion stress, producing major insights into the RAS pathway, heterochromatin, and plasmids. Friday finished with several important talks on evolution. Aashiq Kachroo (The University of Texas at Austin) described efforts to understand the rules governing gene replaceability between yeast, humans, *Escherichia coli*, and plants by systematically rescuing essential gene deletions with orthologous proteins. Joseph Schacherer (University of Strasbourg) described the 1002 Yeast Genomes project, which sequenced > 1000 diverse yeast isolates from natural environments, revealed an unprecedented view of genomic variation, and, coupled with phenotypic studies, permitted genotype–phenotype linkages to be suggested. As yeast usually pushes boundaries in this area, the “Revisiting Classical Genetics with New Technology” session on Saturday was extraordinary. Michael Costanzo (University of Toronto) presented the complete genetic interaction network of *S. cerevisiae*, moving toward the first long-sought wiring diagram of a eukaryotic cell. Zhimin Liu (Laufer Center) described new ultrahigh-throughput protein–protein interaction screening methods, which use deep sequencing of barcoded strains where bait- and prey-associated barcodes can be fused for sequencing of interacting proteins. Uri Weill (Weizmann Institute of Science) described a collection of N-terminal GFP fusion proteins that can be rapidly swapped to any other tag in an automated fashion. Agnes Michel (ETH Zürich) described coupling a saturating transposon–insertion screen with deep sequencing. Remarkably, this could map not only essential genes, but had sufficient resolution to reveal essential protein domains, genetic interacting partners, and drug sensitive or resistant alleles. Finally, Saturday night saw Lars Steinmetz (Stanford University and EMBL) receive the Ira Herskowitz Award, highlighting his important work on pervasive transcription and the technological advances that have led to a redefinition of the complexity of transcriptional isoforms and their potential role in cellular heterogeneity. The last day opened with a session on metabolic regulation (“The Fat and Sweet Sides of Life”), including Kobi Simpson-Lavy (Tel Aviv University), who shed light on the implications of protein aggregation in glucose repression. At the end of the session, poster awards were presented to three outstanding graduate students and one outstanding undergraduate student.

The next Yeast Genetics Meeting will be held in 2018. Stay tuned for more details of the dates and location!

## 12th International Conference on Zebrafish Development and Genetics

A highlight of the meeting was Didier Stainier (Max Planck Institute for Heart and Lung Research) bringing to a close the long, arduous struggle to clone one of the first zebrafish mutants to be described: *cloche*. The fascinating aspect of *cloche* is that its embryos are bloodless and also lack vasculature, meaning that it plays a very early role in the diversification of the early mesoderm into endothelial and hematopoietic tissue fates.

It was recognized long ago that the *cloche* locus occupied a position very close to the distal tip of the telomere on chromosome 13. This region on the zebrafish genome is poorly assembled thereby greatly complicating efforts to identify the underlying gene by classical positional cloning. The pursuit of this locus became the holy grail for several groups.

The approach taken by the Stainier laboratory provides further evidence of the power conferred by CRISPR/Cas9 technology. The researchers reasoned that *cloche* must be expressed prior to gastrulation and so carried out RNA-seq on pools of individually genotyped wild-type and *cloche* mutant embryos at 6–10 h postfertilization to identify significantly downregulated genes. Having identified 19 candidates, 18 gave no phenotype upon mutation using CRISPR. Knocking out the final candidate to be tested, *npas4-like (npas4l)*, phenocopied the mutant. This gene encodes a member of the bHLH-PAS family of transcription factors and directly regulates the *etv* and *tal1* genes, the earliest expressed endothelial and hematopoietic transcription factor genes identified so far. The identification of *cloche*/*npas4l* as a master regulator of endothelial and hematopoietic fate may lead to improved protocols for the generation of endothelia and hematopoietic cells *in vivo* (Reischauer *et al.* 2016).

In another impressive study, Daniel Grimes (Princeton University) utilized zebrafish deficient in the *ptk7* gene, which display a curved spine, reminiscent of human adolescent idiopathic scoliosis (IS). He revealed underlying defects in the formation and function of motile cilia in the central nervous system of this mutant, which perturbed the flow of cerebrospinal fluid (CSF), and caused abnormal spinal curvatures as the fish grew. Restoration of cilia motility after the onset of scoliosis blocked spinal curve progression. The results implicate irregularities in CSF flow as an underlying biological cause of IS, and suggest that noninvasive therapeutic intervention may prevent severe scoliosis (Grimes *et al.* 2016).

Many talks and posters highlighted the use of zebrafish as disease models. Craig Ceol (University of Massachusetts Medical School) showed that Gdf6 expression is upregulated in melanoma, and plays a role in inducing their neural crest cell identity, which is key to their malignant behavior. Marcel den Hoed (Uppsala University) presented a zebrafish model of atherosclerosis and demonstrated that Flt1 protects vasculature in the larvae from lipid deposits. Steve Farber (Carnegie Institution for Science) introduced new tools for studying high cholesterol levels. Tom Carney (IMCB A*STAR) presented work in which the liver in zebrafish is “humanized” by expressing human detoxification enzymes in this organ. This allows zebrafish to be used to more accurately assess toxicity of compounds on the human organ. Finally, in his plenary lecture, Len Zon (HHMI/Children’s Hospital Boston) showcased the value of the zebrafish as an animal model for human disease (for details see summary below).

The Chi-Bien Chien Award was presented to Adam Miller (University of Oregon) for his work on electrical synapse formation. This award is given to a graduate student, postdoctoral associate, or newly appointed faculty member who has made a significant contribution to the zebrafish field. The George Streisinger Award recognizes a senior investigator who has made outstanding and continued contributions to the advancement of the zebrafish field. The inaugural award was given to Chuck Kimmel (University of Oregon) and will be presented at the upcoming Strategic Conference of Zebrafish Investigators in 2017.

### 

#### Other highlights included:

Jessica Nelson (University of Pennsylvania) revealed an unexpected role of huntingtin protein in learning.

Shinsuke Seki (Tokyo University of Marine Science and Technology) used medaka spermatogonia to produce oocytes and sperm when transplanted; it may work for zebrafish too!

Ashley Bruce (University of Toronto) showed that yolk syncytial layer (YSL) nuclei move through the microtubule network to drive epiboly (vegetal movement of the blastoderm and the YSL to enclose the yolk cell). It was previously thought to be driven by depolymerization of the network.

Chase Bryan (University of Utah) produced some amazing movies of optic cup morphogenesis.

Xuefei Yuan (The Hospital of Sick Children) gave a great talk on finding noncoding regions that drive expression in cardiac precursors.

Yahui Lan (Weill Cornell Medical College) showed that zebrafish *tet2/3* mutants have a loss of epicardial migration and proliferation, and highlighted the role of DNA methylation in the process.

Aaron Savage (University of Sheffield) used tissue-specific expression of NLS-Cas9 for tissue-specific knockouts by injecting sgRNA.

Autumn Marsden (University of Iowa) showed that the EF-hand domain in Naked Cuticle (Nkd) is not required for canonical Wnt inhibition, but is required for Wnt-PCP.

Tamara Stawicki (University of Washington) showed that intraflagellar transport genes are important in lateral line hair cell resistance to neomycin.

Rob Cornell (University of Iowa) analyzed a tissue involved in oral-facial development (the periderm) to find genes involved in oral-facial clefting.

The 13th Zebrafish Development and Genetics Meeting will be held in the summer of 2018 in Madison, WI.

## Model Organism Genome Resources

The gathering of so many model organism geneticists in one place made TAGC a natural forum for discussion of issues surrounding the funding and organization of MODs. Immediately before the meeting, several leaders in the community, with logistical support from GSA, wrote a statement of support for the MODs that was signed by over 11,000 model organism geneticists and presented at TAGC to Francis Collins and the National Institutes of Health (http://genestogenomes.org/action-alert-support-model-organism-database-funding/). The statement expressed enthusiastic support for the initiative to integrate elements of the existing MODs, but voiced concern about the possibility that overall support for MODs would not be sustained. Collins directly addressed these concerns in his joint plenary keynote presentation (see summary below).

The meeting also coincided with the launch of the Alliance of Genome Resources (AGR), which was formed by the Gene Ontology Consortium and six MODs: *Saccharomyces* Genome Database, WormBase, FlyBase, Zebrafish Model Organism Database, Mouse Genome Database, and Rat Genome Database. The AGR was created to provide better support for the biological sciences via an integration of shared data, standardization of data models and interfaces, and unified outreach to researchers, educators, and the public (http://genestogenomes.org/model-organism-databases-join-forces-announcing-the-alliance-of-genome-resources/).

## Jef Boeke

### Yeast, the model eukaryote, leads the way in designer genomes

#### Synthesizing and SCRaMbLEing a genome:

For the past 10 years, Jef Boeke (New York University Langone School of Medicine) has led an international group of researchers in an effort to synthesize the yeast genome. Known as *S. cerevisiae* 2.0 (Sc2.0), this “designer” genome will be fully synthesized from oligonucleotides. With support from the National Science Foundation along with additional funding secured by collaborating institutions around the world, this huge undertaking aims to build a yeast genome that will reveal fundamental insights into biology.

To construct Sc2.0, the researchers are using the annotations in the *Saccharomyces* Genome Database (SDG) along with an assembly method called SWAP-IN. The investigators use SWAP-IN to gradually swap pieces of the yeast DNA for pieces of synthetic DNA until they have a designer synthetic chromosome derived completely from oligonucleotides. So far, the genome has been designed, and more than 60% of the yeast genome has been synthesized using this approach.

Noting that designing and synthesizing a genome is not an interpretive exercise like reading a book or a DNA sequence, Boeke said planning an entire genome is more akin to a creative act involving a series of decisions. The researchers decided to modify the yeast genome in ways that would make it more stable, such as by removing repeat elements, introns, and transposons. They also removed all the transfer RNA (tRNA) genes, which are hotspots for genome instability, with a plan to relocate them to a special new chromosome they call a “neochromosome,” being designed by Patrick Cai and colleagues at the University of Edinburgh.

##### Insights on chromosome number:

When tackling chromosome 1, yeast’s smallest chromosome, the researchers were concerned that making it smaller by removing large subtelomeric repeat elements might make the chromosome unstable. This concern, combined with the fact that adding the new neochromosome would result in 17 chromosomes for Sc2.0, led to a decision to fuse chromosome 1 to another chromosome. This was successfully achieved using a CRISPR-based method, with little effect on global function; the yeast still grew well.

After their success in fusing chromosome 1, the team considered other organisms with dramatic variations in chromosome number. For example, “Jack Jumper ant” females have a 2n = 2 karyotype, and since the males are haploid they operate with a single chromosome (Crosland and Crozier 1986). At the other extreme is a fern with hundreds to > 1000 chromosomes. Since they had successfully reduced the budding yeast chromosome number from 16 to 15, the researchers pondered how much further they could go. Thus far, they have fused several chromosomes, with no serious fitness defects resulting from these fusions.

Following up on earlier work by Neurohr *et al.* (2011), which enabled construction of a chromosome one-quarter the size of the yeast genome, the researchers are now working to string even more chromosomes together. They aim to first produce a yeast genome with four chromosomes of roughly equal size. If this can be accomplished, they will attempt to see whether the entire genome can reside on just one chromosome. If they are not able to reduce the number of chromosomes this much, it could help reveal factors underlying differences in chromosome number between species.

##### Ribosomal DNA and lessons on 3-D chromosome structure:

Ribosomal DNA (rDNA) resides on chromosome 12. Junbiao Dai and colleagues from Tsinghua University built a synthetic chromosome 12 that retained the yeast rDNA, and then removed the rDNA after supporting the cell’s growth with plasmid-borne rDNA. They were then able to “reprogram” the species identity by altering the internal transcribed spacer sequences of the rDNA. Subsequent experiments demonstrated that moving rDNA to another chromosome did bring about substantial changes in chromatin architecture, but was not associated with large fitness defects.

Collaborators at the Pasteur Institut, led by Romain Koszul, also studied the trajectories and 3-D chromatin structure of the various synthetic chromosomes, finding that the way the synthetic chromosomes are positioned in the nucleus was surprisingly similar to the native counterparts. This suggests that transposable elements do not significantly impact the structure of chromosomes in the nucleus, in yeast at least.

##### Genome scrambling:

The researchers developed a genome scrambling system called SCRaMbLE (synthetic chromosome rearrangement and modification by *LoxP*-mediated evolution) that can be used to create many variants from genomes with one or more synthetic chromosomes. This approach involves inserting symmetrical *LoxP* sites in the 3′ untranslated region of each nonessential gene and at various landmarks, such as the site where a tRNA gene is removed. Once the synthetic chromosome is synthesized and validated structurally, the researchers add a chemically regulated Cre recombinase. When the recombinase is activated, it induces the formation of precisely defined deletions, inversions, translocations, and duplications.

Boeke likened the technique to evolution on steroids. Activating the Cre recombinase in a synthetic or partially synthetic genome yields many millions of variants in one small tube of cells. These distinct “scrambleotypes” give rise to varied phenotypes that can be assessed for desired qualities.

If the genes in the Sc2.0 genome are thought of as a deck of 5000 cards, then SCRaMBLE can be used to delete any combination of cards (genes), change card order, and duplicate or add back extra cards. Because of this capability, genome scrambling can be used to determine the minimal genome possible and to obtain gain-of-function mutations in which the altered gene product possesses a new function or a new pattern of gene expression. Since SCRaMBLE makes sequential changes, it can also be used to explore possible trajectories for evolutionary changes in gene content. SCRaMbLE might even be used to understand how much genome rearrangement it takes to define a new species in the laboratory. It may best be performed in heterozygous diploids, because when used in haploids, deleting genes is more likely to be lethal.

SCRaMBLE was used on the right arm of synthetic chromosome 9, the smallest chromosome arm in the yeast genome, which was synthesized in a circle with 43 segments (Shen *et al.* 2016). Using SCRaMBLE on yeast cells containing this synthetic chromosome arm allowed the researchers to choose random colonies with high fitness. Examining all the different scrambleotypes from the different colonies revealed massive combinatorial diversity. Even with deletions that reduce the length of the chromosome arm by a third, and massive duplications that make it almost four times larger than the original, high fitness was still obtained, and the rest of the genome was unaffected. More recently, the researchers have used genome scrambling to look for desirable phenotypes, such as yeast variants that grow at higher temperatures, which might be useful for brewing, winemaking, and clinical applications.

##### New possibilities with neochromosomes:

**Figure 1:**
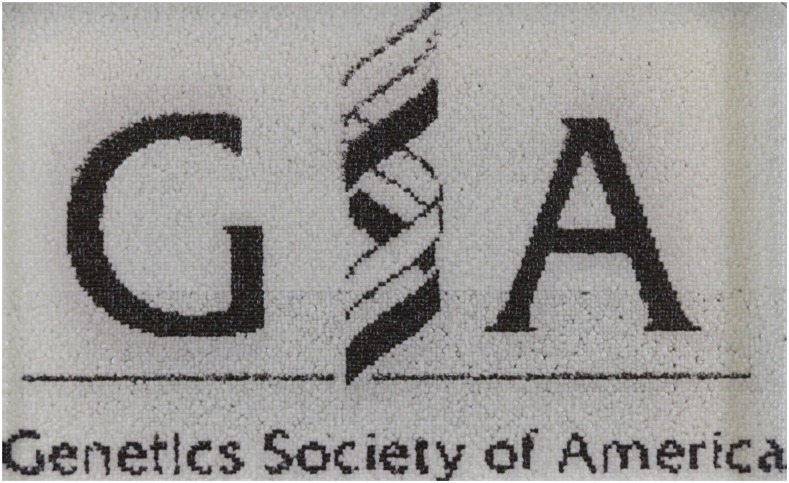
This “living logo” is made up of yeast growing on a rectangular Petri dish. The yeast strains are engineered to produce large amounts of violacein, a pigment from *Chromobacterium violaceum*, leading to a very dark purple that appears black here (Mitchell *et al.* 2015), and wild-type yeast that appear white here. The yeast cells are deposited on the plate using an acoustic droplet ejection robot, creating 25,000 “biopixels.” For more information, see www.yeastart.org.

Neochromosomes are synthetic chromosomes not found in nature. Boeke’s team has used combinatorial neochromosome assembly to create various pigment combinations in yeast and is exploring the use of multicolor yeast for living artwork (see Figure 1). With robotics, they expect to be able to make 100 kb of chromosome every 2 weeks. Boeke is a founder and director of Neochromosome, Inc., through which he intends to commercialize neochromosomes assembled in yeast.

##### Moving to mammals:

Wrapping up, Boeke noted that it might be possible to use SWAP-In with mammalian cells. The Genome Project-write (GP-write) project is a proposal to develop technology that will reduce the costs of engineering and testing of large genomes more than 1000-fold within 10 years (Boeke *et al.* 2016). While the Human Genome Project aimed to read the human genome, GP-write has the goal of building human and other genomes from scratch. This could enable the synthesis of many mammalian-sized genomes, perhaps thousands or more, which would allow testing of the impact of systematic alternations at a genome scale.

GP-write is being envisioned as an international collaborative project, much like the Sc2.0, with a launch planned for late 2016. In addition to developing new technologies, the project also plans to develop an ethical framework for genome-scale engineering.

## Francis Collins

### Accelerating insights from basic genetics

#### A perspective from NIH Director Francis Collins:

Francis Collins has served as Director of the NIH since 2009. A physician and geneticist, he previously led the International Human Genome Project and was the director of the NIH’s National Human Genome Research Institute (NHGRI) for 15 years. He offered a 30,000-foot view of how the NIH supports basic science, and specifically model organism research, highlighting what challenges NIH is facing, and how it is empowering research across different model organisms to further invigorate and inspire biomedical scientists.

Collins dedicated his talk to the memory of his friend and colleague William Gelbart of Harvard University, a widely respected molecular and cellular biologist who encouraged and empowered others throughout his extraordinary life and career. Among his many accomplishments, Gelbart created FlyBase, the genetic database that serves as an indispensable resource for the *Drosophila* model organism community.

##### NIH support for basic research:

As the largest supporter of biomedical research in the world, the NIH is dedicated to furthering both basic and applied science. Although its stated mission clearly includes both fundamental knowledge and its potential applications, an overall decline in science funding over the past 15 years has led to a misperception that the NIH is prioritizing application-driven research over basic science. Emphasizing that the NIH administration strongly supports basic research, Collins outlined a recent analysis of the NIH’s grant-funding track record that demonstrates this commitment (Collins *et al.* 2016).

**Figure 2:**
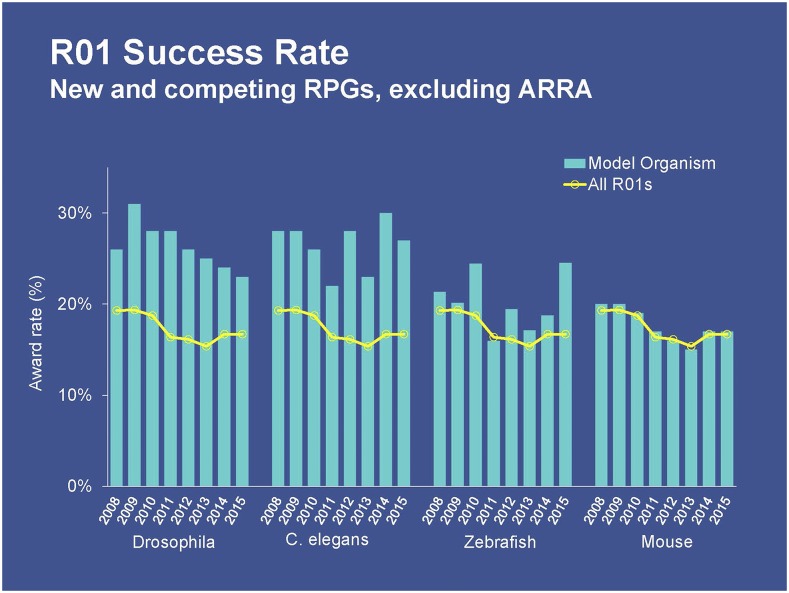
Analyses conducted by the National Institute of Health’s Office of Portfolio Analysis show that the award rates for Research Project Grants (R01s) involving key model organisms have remained steady and, in some cases, are increasing when compared to all R01s. ARRA stands for American Recovery and Reinvestment Act of 2009.

Despite significant budgetary challenges faced by the agency as a whole, Collins said that the NIH remains committed to outstanding research being conducted in the model organism community. In response to a study published in *Genetics*, which reported a decline in NIH funding for *Drosophila* research (Wangler *et al.* 2015), the NIH initiated a follow-up investigation. Using sophisticated search techniques to comb through verified grant records (as opposed to the simple keyword searches used by Wangler *et al.* (2015), an extensive analysis by the NIH’s Office of Portfolio Analysis (OPA) concluded that there had been, in fact, no reduction in *Drosophila* funding (Figure 2).

According to Collins, the OPA found similar results in its analyses of NIH support for research involving other model organisms. For example, *C. elegan*s funding has remained steady, and investigator-initiated research (R01) grants for *Danio rerio* (zebrafish) are actually increasing. The OPA also analyzed R01 success rates to determine whether there could be a bias related to the scientific peer-review process. In fact, the results suggest the opposite: grant success rates are substantially higher for model organism research involving *Drosophila*, *C. elegans*, and zebrafish.

Research trends for mouse models are more difficult to parse, but Collins noted that almost half of all R01 projects utilize mice, suggesting that funding for research using this key model organism remains robust. Collins pointed attendees to the NIH Office of Extramural Research’s “Open Mike” blog (Lauer 2016) for further discussion of NIH’s support of model organism research.

##### Dealing with big data:

Big data are both an enormous opportunity and an enormous challenge for scientists, and the NIH is devoting tremendous time and energy to wrestling with this issue. Collins cited an in-house estimate indicating that NIH science generated ∼650 petabytes of data in the past year alone, a number that continues to grow rapidly. For comparison, he pointed out that the entire content of the Library of Congress comprises only 3 petabytes of data.

Big data are central to the practice of science today (Bourne *et al.* 2015). An NIH Working Group on Data and Informatics, created in 2012 to examine this issue, recommended emphatically that the NIH confront big data challenges head-on (NIH 2013). To that end, the NIH is crafting a plan to capture and manage research data more effectively, tag it with appropriate metadata, and make it more easily findable and computable by the worldwide research community. One important NIH initiative in this area is the Big Data to Knowledge (BD2K) training and research program, created in 2013 with an annual budget that has been growing to reach $100 million. Philip Bourne was hired as Associate Director of Data Science (ADDS) for the NIH and also coleads the Scientific Data Council. Phil and his ADDS team are currently leading BD2K, and also seeking to assist management of data and informatics priorities across all 27 NIH institutes. Complementing those efforts, the NIH has also recently established a cross-NIH task force in charge of data infrastructure. The task force is seeking ways to pilot deposition of data sets and appropriate analytics in the cloud.

##### Data challenges in the model organism research community:

Like most other areas of biomedical science, model organism research is generating ever-larger data sets as genomic techniques, computational biology, and related research techniques continue to become more sophisticated. In addition, Collins noted an emerging interest in the community: analyzing data sets across various species of model organisms. Therefore, model organism researchers are uniquely positioned to become pioneers in the effort to bridge data management, access, and availability across large and often disparate databases, enabling scientists to follow innovative and unforeseen research paths.

Model organism-specific genome databases came about in the 1990s, alongside the Human Genome Project. Collins said these databases, often funded by the NHGRI, are well designed, useful, and have the full support of the NIH. They are also extremely successful: tens of thousands of researchers use the data, which go well beyond genomics, on a monthly basis. However, each organism-specific database was created as a separate entity, and, as such, they lack shared standards that could further encourage cross-species analyses. Therefore, the NIH envisions a reorganization of the existing model organism databases (Mouse Genome Informatics, WormBase, ZFIN: The Zebrafish Model Organism Database, *Saccharomyces* Genome Database, FlyBase, and the Gene Ontology Consortium) to open them up to a wider community and facilitate more cross-organism investigations.

According to Collins, such a change would offer many benefits for science. For example, a search for human orthologs in different model organisms currently requires a researcher to run individual organism searches, analyze results, and perform calculations on each database separately, and then attempt data integration. That is a very complex process to answer a fundamental and valuable research question. Collins said tackling the same question with a more unified and standardized genomic database that spans multiple model organisms would be much more efficient and could produce more reliable results.

Another reason for integrating these databases is that the cost of hosting ever-growing stores of data on individual servers is increasingly straining the NIH’s budget, whereas cloud-based storage alternatives could enable a more sustainable funding model across genome databases. Cloud computing also can make data analysis simpler, removing the slow download times that large data sets require in order for analyses to be performed. Toward this end, BD2K is currently working to establish the NIH Commons, a cloud-based virtual ecosystem of indexed, accessible biomedical data. The NIH Commons will widen the audience for powerful data stores, while at the same time simplifying their use to increase the ease and efficiency of scientific inquiry.

MODs would make excellent pilot databases for the Commons, Collins suggested, noting that the NHGRI has met with the MODs’ principal investigators to exchange ideas on the proposed integration. As an outgrowth of those discussions, the AGR has been formed and tasked with the challenging goal of charting a plan for integrating the MODs. To help achieve this bold vision, Collins said those running each MOD will need to update data management practices, enhance the interoperability of data and the integration of content, commit to reducing redundancies, and share best practices.

While many scientists see value in such a reorganization, some remain worried that such an effort could degrade these critical resources. Collins pointed to a recent letter to the NIH signed by more than 10,000 model organism researchers that endorsed a general need for database integration, but also expressed concerns about the speed of the process and the need to retain certain essential functions of the individual databases. Stating that the NIH takes such concerns very seriously, Collins emphasized that the agency is taking great pains to preserve the value and services that each database currently provides, and does not intend to rush what is unquestionably going to be a complex process. Collins stressed, however, that maintaining the status quo is not an option: individual databases must be made interoperable to create a single, federated view of model organism data that will be useful, useable, and sustainable.

##### Bolstering public support for basic research:

Wrapping up, Collins reflected on the very tight fiscal climate within which the NIH—and the biomedical research community that it supports—has been operating since the early 2000s. These have been lean years, he said, and the constrained budget is reflected in today’s historically low success rates for grant applications. However, Collins said that he sees light at the end of the tunnel: The NIH budget for Fiscal Year (FY) 2016 included an increase of $2 billion, a 6.6% increment that was made possible by bipartisan Congressional support. Likewise, Collins noted that the FY2017 House and Senate budget proposals for the NIH also look promising.

Collins urged attendees to consider their own roles in making the case to Congress and to the American taxpayer for the value of biomedical research. Basic science needs champions to tell the world how essential such work is, how remarkable the findings are, and how such discoveries can serve to advance medicine and stimulate the economy. Indeed, Collins said model organism researchers should seize this opportunity to become a symphony of voices sharing their excitement over their work and its potential to change the world.

## Jennifer Doudna

### CRISPR-Cas genome engineering

#### The biology, technology, and ethics of a remarkable technique:

Ever since the discovery of the double helix structure of DNA, scientists have wondered if it would be possible to manipulate genomes, perhaps even at the level of individual base pairs. Jennifer Doudna (University of California, Berkeley) summarized her work in the development of CRISPR-Cas9, a technology that is ready to do just that.

Although Doudna is today perhaps best known for her contributions to CRISPR genome editing, her career has focused not on DNA, but on RNA structures. Doudna recounted how the biochemistry of CRISPR systems and CRISPR-associated genes first came to be understood, how the gene editing technology was then developed, and what ethical implications are arising as the field moves forward.

##### Insights into bacteria–virus interactions:

The CRISPR story starts with bacteria. Bacteria live in constant battle with viruses, and so they have evolved many different ways to fight off viral infections. One of the ways is through “memorizing” sequences of viral DNA. In the context of studying these mechanisms, Doudna was introduced by Jill Banfield, a colleague at UC Berkeley, to the phenomenon of CRISPR, which are repetitive sequences of DNA in a bacterial genome. These repetitions flank short DNA sequences, 30–40 bp long, that originally come from invading viruses. CRISPR evolved as a method for bacterial cells to maintain a genetic record of viruses that have infected them, while also passing on that record to future generations of bacteria. In addition, specific “*cas*” (CRISPR-associated) genes are located near these sequences, which suggested involvement of a conserved pathway.

In subsequent studies, Doudna and her colleagues further deciphered the biochemistry of this process. CRISPR-Cas proteins can detect foreign DNA in a cell (such as that from a virus) and integrate small sequences of that foreign DNA into the bacterial CRISPR array. These arrays become RNA copies that then combine with Cas proteins to become DNA “detectives,” searching for viruses by finding foreign DNA that matches the RNA transcripts. If found, the viral DNA is bound, cut, and degraded. This process illustrated an important way that bacteria cleverly adapt to viral invaders, and also provided valuable clues for a new approach to altering the genome.

##### Tracing the role of RNA:

Doudna’s work had long focused on how RNA controls gene expression in cells, and especially how it can recognize other nucleic acids. This pursuit led her to a collaboration with Emmanuelle Charpentier (Max Planck Institute for Infection Biology). Together, Doudna and Charpentier identified the biochemical activity of one Cas enzyme, named “Cas9” (CRISPR-associated protein 9).

Further study revealed aspects of Cas9 that make it truly remarkable (Jinek *et al.* 2012); it can bind to double-stranded DNA by matching a 20-nucleotide guide sequence in an RNA molecule. This reaction requires a second RNA molecule, known as the “tracr” (*trans*-activating CRISPR RNA), which interacts with the end of the CRISPR RNA enabling Cas9 to easily bind. This dual RNA-guided protein is what provides the immunity from viruses that previous studies had found but not completely explained.

##### Learning to harness the power of Cas9:

Understanding the mechanisms behind Cas9 suggested it might be harnessed to serve as a tool for genome editing. It could even be simplified; Doudna’s RNA expertise enabled her to connect the two RNA molecules and create one single RNA guide that would contain both the “handle” for Cas9 to bind and the DNA-targeting sequence. The combination is easily programmed to recognize a desired DNA sequence and make a targeted double-strand break.

Doudna and her team developed a 3-D structural model to illustrate the details. Cas9 attaches to the guide RNA, and together they recognize the DNA sequence, unwinding the double helix to allow the RNA to bind and form an RNA/DNA hybrid that displaces the other DNA strand. Finally, Cas9 makes breaks in each DNA strand at the targeted sequence.

Cas9 comes on the heels of a number of previous gene editing technologies, including zinc finger nucleases and transcription activator-like effector nucleases. These methods also introduce double-strand breaks at specific sequences, triggering DNA repair via either the nonhomologous end joining pathway or homology-directed repair. Although they are effective, engineering and testing these proteins is extremely expensive and time-intensive. By contrast, Cas9 can be easily and quickly programmed, a vast improvement over the previous methods.

As their understanding of Cas9 grew more detailed, Doudna and her team remained stymied by one question: how does Cas9 unwind the DNA strands without an external energy source? Through comparisons of crystallographic images of Cas9 at work, they found that the protein radically changes its conformation as it forms a path with the guide RNA. It then binds to the DNA and changes again, pushing away one DNA strand as the other strand base-pairs, or “zips together” with the RNA guide. Follow-up studies using fluorescent markers confirmed these structural changes and pointed to their role in allowing the protein to only cut DNA that is a perfect, or near perfect, match to the guide RNA. The team also discovered that the search for DNA is three-dimensional: the protein does not bind once and slide along the strand until finds a match, but rapidly binds and releases as it searches for the target sequence (Knight *et al.* 2015).

##### The rapid rise of CRISPR-Cas9:

Though there are still more details to be fully uncovered, the CRISPR-Cas9 gene editing system has worked remarkably well in the many cell types in which it has been tested and, as a result, has been rapidly adopted in many fields.

Doudna offered three reasons for the success and rapid rise of CRISPR-Cas9. First, the recognition mechanism relies on RNA/DNA base pairs, not a protein–DNA interaction, so it is much easier to program and change, akin to programming software rather than hardware. Second, CRISPR-Cas9 technology became available at the same time that researchers were learning much more about the role of genes in disease, and could therefore envision a range of enticing applications for the process in medicine and other fields. Finally, thanks to its natural evolution in bacteria, CRISPR-Cas9 is fast and accurate when detecting target DNA sequences.

##### Future directions and ethical considerations:

Despite its success so far, Doudna spoke of three key challenges to genome editing via CRISPR-Cas9: delivering it to the necessary cells or tissue, controlling the repairs, and considering the ethics of potential applications. This last challenge is especially important when considering the potential use of CRISPR-Cas9 to alter a human genome.

Doudna’s lab so far has focused on the biochemistry involved, and specifically the question of how to use what we know so far about CRISPR-Cas9 in order to deliver it to different types of cells. To that end, the team is currently testing the delivery of CRISPR-Cas9 and guide RNA as a preassembled, purified protein/RNA combination. Results for human T cells and mouse brain cells so far have been promising and suggest that CRISPR-Cas9 could eventually be used to treat various genetic disorders.

The ethical implications of this technology deserve careful scrutiny, and care must be taken to ensure that CRISPR-Cas9 is applied for biomedical, agricultural, and environmental advances in ways that are both safe and ethically sound. To this end, Doudna asserted that there is an urgent need for informed scientists to engage in broader discussions about the wider ethical and societal implications of this work.

## Anna-Katerina Hadjantonakis

### Single cells get together: cell lineage specification and tissue morphogenesis in the early mouse embryo

#### Challenging the dogma about tissue segregation:

Anna-Katerina Hadjantonakis (Memorial Sloan Kettering Cancer Center) seeks to visualize the complexities of cell behavior in living mouse embryos. By doing so, her team want to understand how tissues are built. Their experiments have revealed a previously unknown role for extraembryonic visceral endoderm cells as the gut tissue develops in the mouse embryo.

These findings challenge a long-held dogma in mammalian development that there is a strict segregation of embryonic and extraembryonic tissue. What Hadjantonakis and her team have seen instead is that some cells derived from the visceral endoderm, which begin in extraembryonic areas, persist in the embryo and contribute to the development of the future gut.

The presentation honored renowned developmental biologist Rosa Beddington; much of Hadjantonakis’ work builds from Beddington’s legacy. It was Beddington who first alerted the field to the importance of the visceral endoderm.

##### Mammalian embryonic development:

For context, Hadjantonakis provided an overview of the currently accepted understanding of embryonic development in mammals, and specifically mouse embryos in the pregastrulation and gastrulation phases. The blastocyst, a universal stage of early mammalian development, has three cell types: the epiblast, trophoblast, and primitive endoderm. As the blastocyst grows, multiple interactions between these three cell types are required to determine the specification and position of their derivative tissue layers. This crosstalk is essential to successful mammalian development.

As embryo development continues, the trophoblast cells form the placenta, the primitive endoderm cells form the amniotic sac, and the epiblast cells form somatic cells which comprise the three germ layers of the developing embryo (the endoderm, mesoderm, and ectoderm). Put another way, it is thought that the trophoblast and primitive endoderm form extraembryonic tissue but do not form any part of the mouse embryo. Rather, the epiblast forms all embryonic tissue, and thus gives rise to all of the body’s somatic cells as well as germ cells.

In contrast, Hadjantonakis’ research suggests that descendants of the primitive endoderm, which later on in development are known as the visceral endoderm, may in fact give rise to some somatic cells, specifically the lining of the gastrointestinal tract.

Breaking the symmetry of the blastocyst is a necessary process during development that leads to anterior-posterior (namely head-to-tail) polarity in the embryo. One aspect of mice that sets them apart from model organisms such as Drosophila or zebrafish is that anterior-posterior polarity is not encoded in the egg. Instead, specialized movements within the visceral endoderm break symmetry via a unique collective cell migration within the epithelium. Beddington’s work, and that of her many collaborators, set the stage for this realization.

##### Insights on cell movements and the role of extraembryonic tissue during gastrulation:

Hadjantonakis and her colleagues sought to visualize cells in living mouse embryos in order to reveal the intricate cell behaviors of gastrulation. To accomplish this goal, her team optimized techniques for culturing mouse embryos *ex utero*; for 3-D, and time-lapse imaging in the embryos; and for studying generational reporters. They also used GFP as a lineage tracer to identify cells as the embryo develops. In the course of this work, Hadjantonakis’ team learned that gut endoderm, though it becomes internal tissue, forms first on the surface on the embryo during gastrulation, which makes its behavior fairly easy to observe.

After the visceral endoderm moves to the anterior of the cup-shaped mouse embryo, the posterior of the embryo begins to form. Then, cells from the epiblast move to the primitive streak (a transient structure which marks the posterior of the epiblast tissue layer), transition, and migrate away as they become mesoderm cells. As gastrulation proceeds, mesoderm cells circumnavigate the space between the two tissue layers, the outer visceral endoderm, and the inner epiblast.

Previously, researchers believed that as epiblast cells came through the anterior region of the primitive streak they became definitive endoderm and in doing so displaced visceral endoderm cells to extraembryonic regions, so that ultimately, there were no visceral endoderm-derived cells in the vicinity of the gut endoderm. But Hadjantonakis’ results suggested otherwise: cells were not displaced. Instead, they stayed put but were dispersed, being incorporated into the gut endoderm as it formed.

Hadjantonakis’ team repeated the experiment with different gene reporters and found this same pattern. An identical distribution of cells also occurred in a subsequent fate mapping experiment, and also when the team generated tetraploid chimeras in which tetraploid cells were labeled with a ubiquitous reporter.

These findings reveal that nascent gut endoderm is made of two different kinds of cells: epiblast-derived definitive endoderm cells and visceral endoderm cells.

##### Investigating the lineage between extraembryonic and somatic tissue in adult mammals:

Hadjantonakis’ group next investigated a link between the extraembryonic tissue (specifically the visceral endoderm) and somatic tissue (specifically the endoderm), which goes on to form the lining of the digestive system in adult mammals.

When the team observed the changes during gastrulation, they saw cells change shape, move from the primitive streak along with the mesoderm, and appear on the surface of the embryo. Eventually, they found that visceral endoderm cells were *not* dispersed in embryos where mutations affected gastrulation.

To understand this process, the team zeroed in on *Sox17*, a gene encoding a transcription factor which is expressed in cells destined to become gut endoderm. *Sox17* is eventually expressed by all cells on the embryo's surface, irrespective of their origin. The researchers were able to conclude that visceral endoderm cells essentially change their identity, becoming the equivalent of epiblast-derived (definitive) endoderm cells, all of which express *Sox17*.

##### The role of Sox17 in cellular identity and endoderm formation:

Cells originating in the primitive streak that become definitive endoderm travel along the mesoderm and intercalate to the outer layer of an embryo. The group hypothesized that this must be how they acquire apicobasal polarity, and further, that visceral endoderm cells must transiently relax this polarity to allow cells into the epithelium.

Given that gastrulation also propels the formation of two basement membranes from just one, Hadjantonakis’ team further hypothesized that perhaps the mesoderm layer cuts this basement membrane in two, or migrates and creates the second layer (a *de novo* formation). Preliminary data from their studies points to the latter.

Interestingly, *Sox17* mutants have a only one basement membrane instead of two, as they have a complete absence of a basement membrane where the mesoderm and endoderm meet. *Sox17* mutants also show a failure of definitive endoderm specification. These findings suggest that *Sox17* must be regulating basement membrane gene expression but preliminary findings suggest that transcription regulation may not be the key mechanism involved.

Hadjantonakis said the team’s results are starting to support a new model of gut endoderm construction: Cells exhibiting a propensity to form endoderm traveling through the mesodermal layer gain polarity, intercalate into the visceral endoderm layer, and make a new basement membrane as they do so. Other genes, which encode basement membrane components, are also critical, and the failure to make a basement membrane causes a cessation of gut endoderm development.

##### The fate of traveling visceral endoderm cells:

When Hadjantonakis and her team used time-lapse imaging of embryo growth, they saw that visceral endoderm derivative cells persist and proliferate after endoderm formation is complete. Although these cells are part of the epithelium, they are very active. One mystery is the exact fate of these visceral endoderm cells. Are they merely a scaffold to bring definitive endoderm cells to the surface of the embryo, or do they persist and contribute descendants to gut endoderm tissues such as the respiratory and digestive tracts and their associated organs? The team is currently investigating this question.

Hadjantonakis’ team is attempting to determine whether there are any visceral endoderm derivative cells along the path in the body from the esophagus to the colon (known as the foregut, midgut, and hindgut) at later developmental and adult stages. Results so far suggest that visceral endoderm derivative cells make up ∼20% of gut tube midgestation cells, with most in the hindgut area (which will become the colon and the rectum).

Hadjantonakis’ previous experimental methods reached their technical limits at a 8.75-d-old mouse embryo. To go further, the team has switched to a more technically challenging process called genetic inducible fate mapping. This work is ongoing, but preliminary data shows visceral endoderm derivative cells are present in the liver, pancreas, and intestine even in a 16.5-d-old embryo.

## David Kingsley

### Fishing for the secrets of stickleback and human evolution

#### The regulatory changes behind evolutionary differences:

David Kingsley (HHMI/Stanford University), presented studies examining evolution in three-spine stickleback fish. Using genetic mapping, Kingsley and his colleagues have identified genes and chromosome regions responsible for a range of evolutionary traits in this species. This research provides strong evidence that regulatory changes in key developmental genes produce classic evolutionary differences in nature. The lessons learned about the genetic mechanisms that underlie the diversity and traits in sticklebacks likely apply to many animals, including humans.

The researchers set out to attempt to answer several questions about evolution:

1. How many genetic changes are required to produce differences seen in nature?
2. What types of genes are involved?
3. What types of mutations occur in those genes?
4. If evolution has a problem to solve, are there lots of different ways to solve it, or does nature tend to use the same mechanisms over and over again?

##### Why study sticklebacks?:

The three-spine stickleback offers an ideal species for studying evolution because it has undergone one of the most dramatic, recent, and repeated radiations in vertebrates. Marine fish established new freshwater populations at the end of the last ice age. New populations subsequently have had ∼10,000 years to adapt to new water conditions, food sources, predators, and parasites. This has produced huge differences in morphology, physiology, and behavior among many independent freshwater populations. Although many of these fish were originally classified as different species, the reproductive barriers between recently evolved forms can still be overcome using artificial fertilization in the laboratory. The genetic architecture of evolutionary differences can thus be mapped in this system, using large families to find the chromosome regions controlling different traits.

When researchers first became interested in studying the molecular basis of evolution in these fish, most of the necessary genetic and genomic tools did not exist. There were almost no established gene sequences, clone libraries, genetic markers, linkage maps, or transgenic methods for sticklebacks. Over the past 15 years, the team has made tremendous progress in developing the necessary genetic, genomic, and transgenic methods. Today, there are complete physical and genetic maps for sticklebacks, a high-quality reference genome, resequencing of many wild populations, and efficient methods for making transgenic or knockout sticklebacks. By combining these molecular methods with traditional genetic crosses, it is now possible to map the key chromosome regions that control major evolutionary differences, and to identify the genes and mutations that influence a variety of traits.

##### A look at limb loss:

Kingsley first focused on studying limb development and pelvic loss, a classic trait that has evolved repeatedly in mammals, amphibian, reptiles, and fish. Marine sticklebacks have pelvic hind fins while some freshwater sticklebacks have lost these fins. By mapping this trait in the sticklebacks, the researchers found that the well-known developmental transcription factor PITX1 is the major locus controlling two-thirds of the variance in pelvic size. The freshwater fish have conserved the amino acid sequence found in the marine fish but have lost expression of it in the pelvis because they lack a tissue-specific enhancer sequence.

The researchers successfully reversed this evolutionary change by reintroducing the marine pelvic enhancer and the *Pitx1* gene into eggs from a stickleback population without hind fins (Chan *et al.* 2010). Introducing a single gene and regulatory sequence was sufficient to bring the pelvic hind fins back to pelvis-less fish. Having confirmed the sequence involved, the researchers compared its evolution in various stickleback populations and found evidence that this trait evolved via independent mutations that deleted this regulatory region.

Taken together, these experiments revealed that a few chromosome regions can have large effects and that the major gene involved in pelvic hind fin loss is a key developmental control gene required for the formation of many different tissues. Even though the *Pitx1* gene plays essential roles in normal development, regulatory changes within the tissue-specific enhancers surrounding the gene can confine the major effects to a particular place in the body.

##### Mechanisms at play in other stickleback traits:

Using the same genetic mapping approach, the researchers identified chromosome regions and genes that control other stickleback traits. Most freshwater sticklebacks have only a few armor plates, while marine species are covered with armor from head to tail. The researchers found that the *EDA* gene—which encodes a signaling molecule in the tumor necrosis factor family—was the major locus controlling 75% of variance in plate number between marine and freshwater forms. In humans, mutations in *EDA* cause human ectodermal dysplasia. In sticklebacks, similar to *Pitx1*, there is a regulatory change in an enhancer that alters the expression of ectodysplasin along the sides of fish to bring about differences in the number of armor plates (O’Brown *et al.* 2015).

Using crosses between white and black sticklebacks, the researchers identified a single chromosome region that controls 50% of the variance in pigmentation score. The primary locus affecting this trait encodes a famous developmental signal in mammals known as stem cell factor, or KITLG. Mutations in this gene produce white mice that are sterile and have severe anemia. The sticklebacks have regulatory alterations that confine KITLG changes to the developing surface structures (Miller *et al.* 2007).

When researchers similarly examined tooth number, armor plate size, and the number and length of spines, they found that in each case, the traits mapped to a few large-effect quantitative trait loci (QTL) or to QTL with small effects. The QTL controlling 20–75% of variance in these traits are all essential signals or transcription factors.

All of the genes identified are also required for normal development. In each case, the researchers found that natural populations have gained or lost enhancers that produce large tissue-specific effects.

##### Broader implications for understanding evolution:

Since the same loci are repeatedly implicated in evolving the same trait in different stickleback populations, the researchers sought to determine whether these mechanisms might extend to other species, including humans. They examined manatees as a mammalian example of pelvic reduction, finding indirect evidence that the *Pitx1* gene might also be involved in pelvic reduction in these marine mammals. They are now conducting genetic studies of manatees, mice, humans, dolphins, cows, and elephants to better understand the mechanisms involved in pelvic reduction.

Genome-wide association studies (GWAS) of classic human pigmentation traits conducted by other researchers showed that human pigmentation differences are associated with the same *KITLG* gene seen in stickleback pigmentation studies (Sulem *et al.* 2007). The human differences also appeared to be regulatory, with the peak association signal mapping more than 300 kb upstream from the *KITLG* transcription unit. Kingsley’s lab used transgenic mice to functionally screen the blond hair association interval in northern Europeans, finding that the human genome contained a tissue-specific enhancer for hair follicle expression in this region. Blonds have a single base pair change in this enhancer that is not present in brunettes. Kingsley’s group introduced the human blond or brunette enhancer into two matched lines of mice and found that a single base pair change is sufficient to lighten hair color *in vivo* (Guenther *et al.* 2014) This work illustrates how eye and hair color can be controlled independently in different parts of the body. For example, altering an enhancer that affects *KITLG* expression in hair follicles but not in the eyes confines the pigmentation effects to just the hair.

Kingsley noted that although pelvis and pigment traits are very different from each other, they hold important implications for understanding evolution since both traits are regulated by an essential developmental control gene surrounded by large regions of noncoding DNA containing many tissue-specific enhancers. In both cases, enhancers within the noncoding DNA control expression in specific places in the body. Changing enhancers in key developmental genes can produce large effects, but these effects are confined to particular anatomical locations, avoiding the deleterious consequences of protein-coding region mutations in the same genes.

##### Looking for patterns:

In their recent work, Kingsley’s group have moved from studying specific cases of trait evolution to using large-scale sequencing of many different stickleback species to find general patterns involved in evolution. Having seen that the same genes are reused when the same traits evolve in different lakes and streams, they analyzed WGSs of many independent marine and freshwater fish to look for other key genomic regions that are repeatedly selected during parallel evolution. The researchers made windows across the genome, and quantified the difference between marine and freshwater sticklebacks compared to variation within the two types (Jones *et al.* 2012). Using this approach, they could see recurrent differentiation between the two stickleback forms for several of the loci they had already identified by forward genetic approaches, such as *EDA*. They also identified 84 regions involved in trait evolution, which they classified as coding for phenotypes, falling between genes, or only showing changes in noncoding DNA. From this, the researchers conclude that the majority of adaptive loci in these repeatedly evolving fish are based on regulatory changes.

Kingsley noted that human geneticists have also identified many regions in the human genome that show signatures of positive selection during recent human history. These genomic regions show very similar patterns to those found underlying stickleback evolution. A fraction of adaptive alleles are based on protein-coding region changes. However, the vast majority of recent adaptive evolution appears to be regulatory in nature in both sticklebacks and humans. Given the similar results across these two very different organisms, similar trends will likely apply to many other complex animals as well.

## Laura Landweber and Richard Miller

### Genome rearrangement and organization in Oxytricha

#### A complex epigenome:

Principal Investigator Laura Landweber and graduate student Richard Miller (Columbia University and Princeton University) presented research on the evolution of complex genomes, using the scrambled and fragmented genome of the ciliate *Oxytricha* as a focal point. The team seeks to understand how *Oxytricha*’*s* genes become reordered from many dispersed parts, how these genes became scrambled over evolutionary time, and the mechanisms orchestrating the events that lead to genome remodeling.

Ciliates have two types of nuclei: a micronucleus, which is the germline nucleus, and a macronucleus, which is for general cell regulation (somatic nucleus). Even though the micronucleus is smaller, it has a complex genome that contains around a billion base pairs—a size and complexity similar to the human genome.

Some ciliates, including *Oxytricha trifallax*, take the complex micronuclear genome and whittle it down by eliminating nearly all of the noncoding DNA. The macronuclear genome can be as small as 50,000–75,000 kb, representing a 90–95% DNA loss compared to the micronuclear genome. Coding DNA regions in the macronucleus can be ordered differently compared to the micronucleus, and must be unscrambled during development of the macronucleus.

##### The genomes of the micronucleus and macronucleus:

The team has gained insights into how genomes are assembled and the epigenetic influences involved in this process by comparing the micronucleus and macronucleus in *O. trifallax*. They discovered that the organism’s macronucleus includes over 16,000 nanochromosomes, most of which code for a single gene (Swart *et al.* 2013). These are flanked by short regulatory sequences and packaged with their own telomeres that mark and protect the sequence ends. There is an average of about 2000 copies of each chromosome and 64 million telomeres per somatic nucleus. All these chromosome copies and telomeres make the macronucleus larger, even though it has a less complex genome.

**Figure 3:**
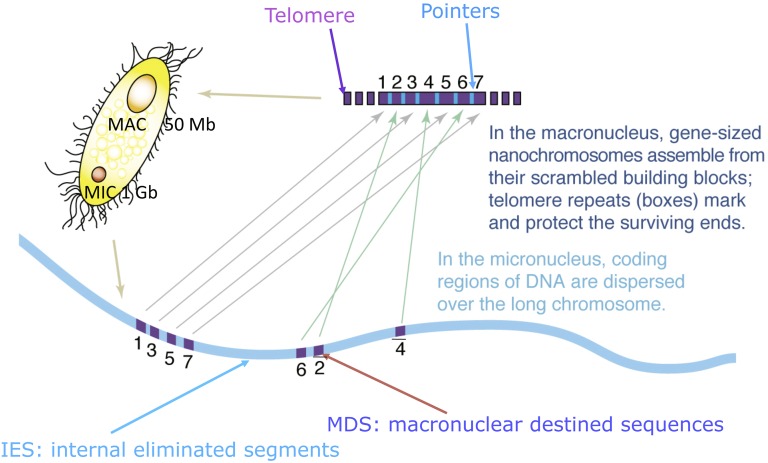
The relationship between a schematic micronuclear (MIC) gene, scrambled during evolution, and its macronuclear (MAC) form, unscrambled during development. Large regions of nongenic sequence (light blue) are removed during development, such that gene segments (purple) are precisely reassembled. Reprinted from Doak *et al.* (2003), with permission from Elsevier.

When the researchers sequenced the germline micronuclear genome, they found more than 225,000 macronuclear-destined sequences (Chen *et al.* 2014). In times of stress, the cells can mate and exchange haploid micronuclei. The intervening noncoding sequences are then removed and the macronuclear-destined sequences put into the correct order (Figure 3) through a template-directed rearrangement process to rebuild a new somatic macronucleus.

Some genes’ precursor loci are combined and condensed in the micronucleus. For example, one germline chromosomal region can give rise to five different genes in the macronucleus while sharing the same first four segments in the micronucleus. All of these somatic chromosomes share the same 5′ subtelomeric DNA but have different gene regulation patterns (Chen *et al.* 2014). There are also short repeat sequences present at the ends (Pointers in Figure 3). These microhomologies may provide guides to assist joining the segments and participate in a process that might be analogous to nonhomologous end joining. Since the short repeats are often just 2 bp, they do not contain all the necessary information for this process.

##### Rearranging scrambled genes:

To better understand the process of DNA deletion and reorganization during macronuclear development, the researchers previously examined the time course of programmed DNA rearrangement for a few scrambled genes. In one case, the precursor DNA has segments 3–8 in order, piece 8 is followed by segment 10, then −2 and −1, and finally segment 9. The first step fuses pieces 3–8 and eliminates the noncoding sequences between them. This first step was found to be one of the most error prone steps, leading to further investigations to identify whether there might be an RNA or DNA guided proofreading step involved. Also during the first step, −1 and −2 are fused, simplifying the locus into a four-segment scrambled gene. Then piece 9 can be merged either between piece 8 and 10, or pieces 9, −1, and −2 can be translated to the end of the gene. This puts −1 and −2 in the correct orientation but leaves 9 inverted. Piece 9 then translocates to produce the final, correctly rearranged product (Möllenbeck *et al.* 2008).

##### Retaining protein-coding sequences:

Landweber and colleagues also sequenced the genome of the micronucleus and made gene predictions using RNA-sequencing reads at different developmental time points. They found that not only are IESs (noncoding DNA) removed from the genome, but that protein-coding genes are also eliminated from the micronuclear genome on the way to becoming a macronucleus. Based on homology, these genes were predicted to have some conserved domains, including some predicted to be involved in chromatin structure and methyltransferase activities.

The researchers found 810 genes that reside only in the germline and are expressed only during development, suggesting they may be involved with breaking up the germline and turning it into a soma (Chen *et al.* 2014). The researchers posit that some of the 810 genes may provide unique functions, while others might enhance existing functions. There are interesting parallels in the *Ascaris suum* roundworm and the lamprey, which both have somatic cell lineages that delete large portions of DNA in their germline cells. This happens at an early stage of embryonic development, and both species have two different genomes within one multicellular organism at later stages.

##### Programming novel gene retentions:

The team developed a method to program novel retentions of germline-limited sequences using Piwi-interacting small RNAs (piRNAs) (Fang *et al.* 2012). This method, which has been useful for creating *Oxytricha* knockout mutants, involves injecting a small RNA during the early stages of mating to introduce an insertion mutation that leads to gene knockout. This allows testing for the retention of that IES in the next generation.

Using this approach, the researchers investigated whether single-stranded RNA injection could program the retention of a germline-restricted gene. They first tested it with an uncharacterized gene that had no predicted domains but that was validated with mass spectrometry data. They showed at least partial retention of the IES containing the gene. Sequencing data also showed at least one complete retention of the IES plus the germline-restricted gene.

The researchers then investigated whether somatically retained germline genes can be expressed during vegetative growth (misexpression mutation). They compared total RNA from the retention line to that of the wild-type line and found that DNA retention interferes with developmental control of a germline gene.

Overall, these findings suggest that single-stranded RNA injection can be used to program the retention of germline-limited genes that reside on eliminated sequences; retention of these germline-limited genes is heritable to at least the F2 generation; and these retained genes lose their developmental control in subsequent generations and are transcribed outside of their normal developmental window.

The researchers posit that the normal developmental control of this micronuclear (germline) gene is subverted by moving it to the macronucleus. This could be because the IES contains all of the regulatory sequences needed for that gene to turn on during late development, but since it is typically present only in the transcriptionally silent micronucleus it is not turned on at any other time. Evidence suggests that the retained uncharacterized protein is transcribed during vegetative growth, but there are other germline-restricted genes present in IES regions that could be tested with this method.

## Michael Miller

### Domestication of C. elegans sperm

#### Insights on cell signaling:

Michael Miller (University of Alabama School of Medicine) studies the *C. elegans* adult gonad for insights into how animal cells coordinate their behavior. The transparent epidermis of *C. elegans* makes it easy to observe in live worms the events leading to fertilization, including sperm migration, oocyte growth, and meiotic progression, and gonadal muscle contraction. Miller and his team seek to delineate the signaling mechanisms coordinating these processes and discern their evolutionary origins.

**Figure 4:**
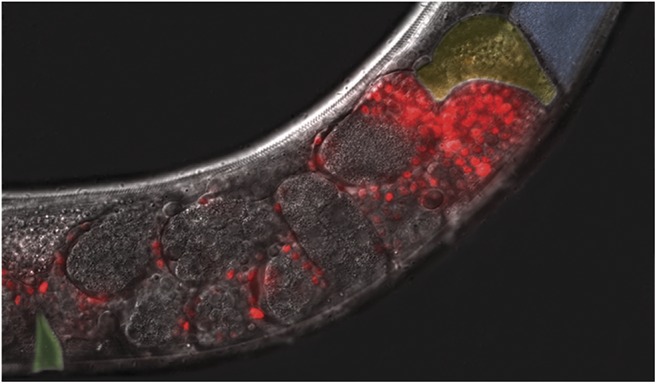
Sperm guidance in the *C. elegans* hermaphrodite reproductive tract. Sperm (red) migrate from the vulva (green pseudocolor) around developing embryos to the spermatheca (yellow pseudocolor). Oocytes (blue pseudocolor) are the source of prostaglandin attractants. Males produce sperm that are more efficient at responding to prostaglandins in specific microbial environments.

In particular, Miller presented research on *C. elegans* sperm, which he described as among the most successful sperm on the planet. In contrast to human sperm, where millions of sperm are present but very few even locate the oocyte, *C. elegans* sperm are thought to fertilize oocytes at a one-to-one ratio. These remarkable sperm have a pseudopod composed of protein fibers that allows them to crawl across the embryos, egg shells, and uterine walls to target the spermatheca where fertilization will occur. About 90% of the sperm in wild-type worms find the spermatheca within 1 hr. One contributor to their efficiency is prostaglandin signals that help guide them to the spermatheca. Previous research suggests that prostaglandins, which derive from the oocytes, provide sperm with positional information and stimulate sperm velocity (Figure 4).

Miller and his colleagues use reverse genetic screens to uncover how these sperm achieve such high efficiency and the role that prostaglandin signaling plays in this sperm guidance process. For the genetic screens, the investigators mate MitoTracker-labeled mutant males with wild-type unstained hermaphrodites and then examine how well the sperm move to the spermatheca. They also use time-lapse imaging to measure velocity and reversal frequency.

##### Prostaglandin signaling:

To test their hypothesis that *C. elegans* sperm use G-protein-coupled receptors (GPCRs) to bind to prostaglandins, Miller’s group conducted genetic screens to look for GPCR mutations that perturb the sperms’ ability to find the spermatheca. These experiments pointed to *srb* chemoreceptors as instrumental in helping sperm respond effectively to prostaglandins. These chemoreceptors are clustered in a 22 kb region of chromosome 2 (Robertson *et al.* 2006). Single-gene and multiple-gene knockouts of the chemoreceptors showed that *srb* mutations affect sperm velocity and reversal frequency. They also found that sperm missing multiple *srbs* showed reduced competitiveness against wild-type sperm. However, mutations in the *srb* genes did not affect fertilization, spermatid size, or activation. In other words, if the sperm with these mutations found the spermatheca, they did not have problems fertilizing the egg. Overall, sperm with *srb* mutations behaved like wild-type sperm, except they did not respond as well to guidance cues.

Contrary to their initial assumption that the srb chemoreceptors were expressed in sperm cells, fusing tdTomato to *srb* genomic loci revealed expression in the ciliated sensory neurons of the male’s nose. Srb mRNA or protein expression was not detected in the gonads. Some of the *srb* chemoreceptors are expressed only in the nose sensory neurons while others have a broader expression pattern, including in the male tail and in the hermaphrodite vulva. The team’s investigation showed that *srb* chemoreceptor signaling is necessary, and sufficient, in nose sensory neurons for sperm guidance. These results indicated that the SRB chemoreceptors are likely responding to cues from the external environment, not from prostaglandins in the hermaphrodite reproductive tract.

##### Oxygen levels and bacterial foes:

The team’s next series of experiments sought to reveal more about how the environment affects sperm motility. They began by examining *srb-13* transcriptome data, which showed a large enrichment for genes involved in immunity and pathogen response. Since this suggested that *srb-13* might be responding to pathogenic bacteria, the researchers tested a panel of 17 microbes known to coinhabit with *C. elegans*. They discovered five bacterial species that increase *srb-13* activity. When these bacteria were grown with the male worms, they produced sperm with poor navigational performance. Males infected with the bacteria that increased *srb-13* activity were smaller, appeared unhealthy, and mated poorly, indicating that the bacteria were likely pathogenic.

The team investigated neuropeptide genes to better understand how SRB-13 transduces signals from sensory neurons to sperm. They found that mutations in *flp-18* and *flp-21* cause sperm guidance defects similar to the ones seen in the *srb* mutants. FLP-18 and FLP-21 are ligands for the neuropeptide Y receptor NPR-1 (Rogers *et al.* 2003), and *npr-1* mutants also showed sperm guidance defects. They also demonstrated that *flp-21* and *srb-13* act via the same genetic pathway.

Since other researchers had shown that NPR-1 suppresses the hyperoxia sensor GCY-35 (Gray *et al.* 2004; Cheung *et al.* 2004), Miller’s group tested the effects of *gcy-35* deletion. They found that *gcy-35* loss suppresses the sperm guidance defects, indicating that *srb-13* mutant guidance defects are dependent on this hyperoxia sensor. Others have shown that GCY-35/GCY-36 heteromers bind oxygen and that at ambient oxygen concentrations GCY-35 activity is stimulated, promoting behaviors important for pathogen avoidance (Gray *et al.* 2004; Cheung *et al.* 2004; Reddy *et al.* 2009). Consistent with this data, growing *srb* mutant males in 10% oxygen suppresses the sperm guidance defects. Taken together, these findings suggest that SRB-13 represses GCY-35 activity.

The researchers believe that wild pathogenic bacteria found in the hypoxic environment inside rotting plant stems trigger SRB-13 chemoreceptor signaling in *C. elegans* gustatory cilia. SRB-13 signaling represses the GCY-35 hyperoxia circuit. GCY-35, in turn, modulates oxidative metabolism gene expression in the testis, which responds by producing sperm with enhanced attractiveness to prostaglandins. SRB-13 seems to improve sperm motility performance in oxygen-rich environments such as the lab petri dish or on the surface of rotting plant stems. Based on these findings, Miller posits that the worms are trying to sense and avoid these pathogenic bacteria and increase their fertility in surface-like environments, away from the oxygen-depleted pathogen colony.

##### Downstream effects in sperm:

To find out what was happening further downstream in the sperm, the researchers used RNA sequencing data to identify potential sperm genes affected by *srb-13* mutations. *Srb-13* mutants exhibited a strong reduction in mRNAs that are involved in oxidative metabolism. Many of these RNAs were mitochondrial, and several of them encode electron transport chain subunits. Performing quantitative PCR on the mitochondrial genes revealed that several, but not all, of the mitochondrial transcripts were significantly reduced in sperm isolated from *srb-13* mutants. This finding suggests that the sperm guidance defects result in part from reduced sperm mitochondrial RNA stability. Looking closer at these mitochondrial genes, the researchers discovered that a mitochondrial genome deletion affecting mitochondrial RNA levels caused sperm guidance defects similar to those found in the *srb-13* mutants. Additional *srb-13* targets encoded in the nuclear genome, such as the mitochondrial fission mediator *drp-1*, also impact sperm guidance. Thus, SRB signaling appears to affect the sperm’s mitochondria, indirectly improving its ability to respond to guidance cues.

##### Domestication of C. elegans sperm:

Miller and colleagues posit that hermaphrodites are domesticating the *C. elegans* sperm. Through additional studies, they observed that *srb* expression is similar in hermaphrodites and males. They also found that *srb* mutations in hermaphrodites reduce brood size, which they attribute to sperm defects (either decreased sperm production, poor sperm guidance, or both), because the researchers were able to rescue the brood size defects by mating the hermaphrodites with wild-type males.

Further work showed that the N2 Bristol laboratory strain of *C. elegans* has increased basal SRB signaling independent of pathogens. Others have shown that N2 Bristol has acquired mutations in multiple genes, including *npr-1*, that repress the hyperoxia-sensing circuitry (de Bono *et al.* 1998; Rogers *et al.* 2003). Like N2 Bristol, several wild isolates collected from across the world have excellent sperm performance in the lab. On the other hand, wild isolates from Hawaii and California show poor sperm performance, suggesting that SRB activity is polymorphic among natural populations. Excellent sperm performance correlates with the presence of large chromosomal regions thought to have swept through the global population roughly 50–200 yr ago (Andersen *et al.* 2012). The researchers speculate that this sweep may have included mutations that increase basal SRB activity, thereby improving sperm performance in oxygen-rich environments. In this context, hermaphrodites may be shaping sperm performance characteristics important for fertility.

## Molly Przeworski

### The evolution of meiotic recombination

#### Examining the role of PRDM9:

While evolution has produced clearly observable differences between species, there is equally complex diversity among individuals within a species. These heritable differences are influenced by drift, mutation, and recombination, in addition to natural selection. Understanding these factors provides a greater appreciation of and understanding for the processes underlying evolutionary changes.

One such factor, known as meiotic recombination—the DNA shuffling that occurs during cell division—is the research focus of Molly Przeworski (Columbia University).

##### Key questions about meiotic recombination:

Meiotic recombination and factors that influence it have drawn attention from researchers across biological fields in recent years. These mechanisms have attracted long-standing interest from molecular biologists because they play fundamental roles in meiosis. They also have important biomedical implications. For instance, proper chromosomal segmentation is necessary to avoid pregnancy complications such as aneuploidy, which leads to spontaneous miscarriage or severe developmental disabilities.

In another scientific community, evolutionary biologists study meiotic recombination for its role in creating new combinations of alleles on which natural selection can act. Some regions of the human genome have greater recombination rates, known as hotspots. Higher recombination rates lead to a more rapid generation of beneficial allele combinations, resulting in more efficient natural selection. Evolutionary biologists have sought to find out if there are differences in hotspots among individuals, and how these differences might affect evolutionary change in recombination profiles.

Przeworski’s research integrates key underlying questions across molecular and evolutionary biology. Her lab has sought to understand how recombination works across different species, why it operates differently in different species, the evolutionary implications of how recombination is regulated, and how natural selection shapes recombination mechanisms to lead to diverse recombination patterns.

##### A method for identifying patterns of genetic variants:

Fine-scale meiotic recombination was originally studied through sperm typing, but, among other limitations, such methods cannot be scaled to the whole genome. Przeworski instead used a method for studying genetic recombination that looks at patterns of the genetic variants in a sample that result from population processes and recombination events from previous generations.

Through this method, researchers can obtain time-averaged estimates of recombination for the ancestors of the sample, including both males and females. In effect, the approach allows researchers to determine what recombination rates in the past are likely to produce the specific patterns of genetic variation seen today. The method is also practical, facilitating a detailed look at the evolution of recombination without requiring cross-breeding of species in the laboratory; a set of individuals just needs to be resequenced.

##### Tracing the factors that influence recombination:

Applying this method to genetic variation data for the last decade, researchers have achieved a fine-scale resolution and identified the position of more than 30,000 hotspots along the human genome.

In 2004, Przeworski and her colleagues used the method to compare humans to our closest evolutionary relatives, chimpanzees, by determining the locations of hotspots for each species (Ptak *et al.* 2004, 2005; Auton *et al.* 2012) and comparing the two sets. Contrary to an initial belief that there would be similarities in hotspot locations between such closely-linked species, Przeworski’s analysis showed the locations of hotspots were in fact extremely dissimilar between humans and chimps, essentially independently distributed along the genomes of the two species. These results suggested that some or many mechanisms of recombination differ between humans and chimpanzees.

Given this foundational understanding that genetic recombination hotspots vary between species, Przeworski shifted her focus to potential individual differences in hotspots within one species. In a study of human genomes, Przeworski found that different individuals show different sets of recombination hotspots (Coop *et al.* 2008). Studying this phenomenon using a pedigree analysis, Przeworski concluded that this trait variation was heritable. Heritable variation for a trait allows for it to be mapped, which in this case revealed how hotspots are specified in humans and mammals generally.

##### The role of PRDM9:

In 2010, three independent studies were published that identified a specific gene, *Prdm9*, as responsible for specifying the location of recombination hotspots in primates and mice. PRDM9 has three domains, the last of which is a zinc finger that specifies DNA binding and another of which is a SET domain that trimethylates the histone H3K4. Researchers found that this gene is able to specify the location of recombination hotspots by binding DNA, trimethylating H3K4, and eventually recruiting SPO11, a protein that plays a role in double-strand breaks on the genome that, when repaired, result in recombinant products.

When a break occurs in one of two homologs, the allele that breaks receives information from the other allele in the repair process. Certain alleles are more or less likely to recruit PRDM9 (termed “hot” and “cold,” respectively). The hot alleles will experience more frequent breaks on the genome; because the break in the hot allele will be repaired by the cold allele, the cold allele will be overtransmitted and the hot allele will cease to exist at the population level.

This process behind recombination in which hotspots can be lost presents an interesting conundrum: If hotspots lead to their own extinction, then why do they exist in nature? One possible explanation exists in the binding domain of PRDM9. Specifically, if the zinc finger of PRDM9 mutates, it could restore a new set of hotspots.

##### Recombination with and without PRDM9:

Przeworski’s research takes advantage of naturally occurring populations that lack PRDM9 to further understand the influence it has on recombination locations. Specifically, she works with four types of birds: the double-barred finch, zebra finch, and two varieties of long-tailed finches. The extent of genetic variation in these PRDM9-lacking species is similar to the variation seen in humans, chimps, and gorillas, making these birds a useful comparison for hotspot evolution. In the bird species, Przeworski found recombination was concentrated at or near transcription start sites, a direct contrast to patterns seen in mice, where PRDM9 was found to direct recombination to take place away from transcription start sites.

Przeworski’s team also compared hotspot locations between the species of birds. If the placements of hotspots were determined by chance, about three percent of hotspots would be shared across species. But in contrast to what she observed in chimpanzees and humans, which showed independent distributions of hotspots, the zebra and long-tailed finch species shared more than 70% of their hotspots.

When recombination is directed by PRDM9, hotspots are lost through overtransmission of cold alleles, or are lost or changed by alterations to the binding specificity identified in the zinc finger of PRDM9. Research in birds lacking PRDM9 reveals that there is extensive conservation of hotspots in the absence of PRDM9. In these species, hot alleles cannot be easily lost because they have other responsibilities, including serving as transcription initiation sites.

While PRDM9 is believed to direct recombination, little is known about how it acquired this role. Mammals benefit from PRDM9, yet the fact that there are species without the gene shows it is not essential to recombination. In ongoing work, Przeworski and her colleagues are examining potential explanations for this, including possible indirect benefits stemming from PRDM9’s role in moving recombination away from transcription start sites and the possibility that PRDM9 may serve as a defense against selfish genetic elements.

## Pamela Ronald

### Tomorrow’s table: organic farming, genetics, and the future of food

#### Case studies in genetically-engineered crops:

Advancing food security for a growing world population and increasing the sustainability of agricultural practices are among the world’s most urgent goals. Pamela Ronald (University of California, Davis) shared her laboratory’s work on engineering rice for disease resistance and flood tolerance, and offered a perspective on plant genetics and engineered crops more broadly.

##### Disease resistance in rice:

Rice is a staple food for more than half of the world’s population, but every year ∼40% of the global crop is lost to the ravages of pests and disease. Conventional breeding practices have yielded disease-resistant strains of rice that are now commonly grown and eaten, but the exact mechanisms of disease resistance in these plants were not well understood until the 1990s.

Ronald and her team investigated the genetics of both *Xanthomonas oryzae* pv. *oryzae* (“Xoo”) and its host, rice, to isolate genes for resistance. Their experiments built on research by Gurdev Khush and colleagues at the International Rice Research Institute (IRRI), who for years collected wild rice species with agronomically useful phenotypes. One in particular, a strain from Mali, was found to be resistant to virtually all strains of *Xoo*. In 1995, Ronald and her team isolated the gene responsible for this resistance, *Xa21*, and discovered that it encodes a receptor kinase bearing a leucine rich repeat (LRR) motif (Song *et al.* 1995). Subsequent studies revealed a remarkably similar set of receptors across many organisms, including in flies, mice, and *Arabidopsis*.

Having isolated the *Xa21* gene and the LRR-containing kinase, Ronald and her team next tackled identifying the microbial molecule that activates this rice receptor. This eventually led to the discovery of *Xanthomonas raxX*. Ronald's lab demonstrated that mutations in several genes in an *Xoo* operon—*raxST*, *raxA*, and *raxB*—disarmed the microbe's ability to activate the plant’s immune response. They then identified a small open reading frame, called RaxX, just upstream of the RaxSTAB operon. RaxX encodes a small protein with a conserved tyrosine, Y41, which is sulfated by raxST. RaxST is the first example of a bacterial tyrosine sulfotransferase (Han et al. 2012; Pruitt et al. 2015), previously thought to be present only in eukaryotic species.

Ronald noted that tyrosine sulfation is essential for modulating receptor-ligand interactions in many species. In an example well-known to plant biologists, sulfation of the *Sinorhizobium meliloti* Nod factor is required for nodulation of alfalfa. Sulfation is also required for the CCR5 coreceptor to recognize and resist gp120, a subunit of HIV (Gardner *et al.* 2015). Ronald pointed out that a portion of the human population has a nonfunctional allele of CCR5, which makes these individuals immune to HIV.

Ronald also showed that a synthetic sulfated RaxX peptide can activate XA21-mediated immunity, and that *Xoo* strains that can overcome *Xa21* have mutations in *RaxX*. Ronald's current focus is on determining if sulfated RaxX is secreted through the RaxA/RaxB type 1 secretion system.

##### Flood resistance in rice:

Floods are another significant danger for the world’s rice crop. Rice grows well in standing water, but if the plant is submerged for more than three days, almost all varieties will die. In places like Bangladesh, where two-thirds of the population’s average daily calories come from rice, sustained flooding could cause widespread famine. Scientists at IRRI had discovered an ancient rice strain capable of surviving up to two weeks of flooding, but conventional breeding methods were not able to introduce this trait into other varieties without undesirable side effects.

Ronald and collaborators David Mackill and Kenong Xu sought to isolate the gene responsible for flood resistance. Using a map-based cloning strategy, Ronald’s team identified three ethylene response transcription factor (ERF) genes that had been linked, in other organisms, with tolerance to environmental stress. One gene, *sub1A*, was both rapidly upregulated under stress conditions and present only in the ancient flood-tolerant varieties. The team inserted *sub1A* into the rice genome using both genetic engineering and marker assisted breeding (MAS). When submerged, the Sub1 rice varieties yielded three times more grain than conventional varieties.

The new Sub1 variety, developed at IRRI using MAS, was planted by an estimated 5 million farmers in 2015. As the climate changes, many parts of the world are experiencing greater precipitation and severe weather, contributing to increased flooding and posing a substantial danger to the world’s rice crop. Sub1 rice has helped ensure a reliable supply of this essential food under increasingly volatile conditions.

##### Social perceptions and the value of genetically engineered crops:

Ronald noted that her work, which involves introducing rice genes into rice plants, is generally more palatable for consumers than other forms of genetic engineering, such as introducing genes from viruses or bacteria into food crops. However, she noted that sometimes this type of genetic engineering is the most effective approach to enhancing food security and advancing sustainable agriculture.

Ronald cited papaya as one example. More than 25 years ago, the papaya ringspot virus, which could not be controlled with any conventional farming methods, caused farmers to lose almost all of their papaya crop. As the virus spread and farmers grew more desperate, a research team led by local Hawaiian Dennis Gonsalves turned to then-new genetic engineering techniques to help.

The researchers introduced a snippet from the genome of a mild virus strain into the papaya’s genome. Field trials of the new crop yielded 20 times more papaya compared to conventional plants. Two decades later, there is still no conventional or organic treatment that can control papaya ringspot virus. As a result, 80–90% of the papaya grown in Hawaii have been genetically engineered.

Genetic engineering techniques are also saving the eggplant crop in Bangladesh, where caterpillar infestations can ruin this staple. Insecticides are effective but highly toxic, especially for farmers who don't have proper safety gear. Geneticists took advantage of an organic approach—the use of Bt, a bacterial protein that can attack the insect—and snipped the Bt gene from the bacteria and inserted it into the eggplant genome. Field tests in Bangladesh show that eggplant farmers are using far less insecticide, and sometimes none at all.

Often consumers’ main concern is whether genetically engineered food is safe to eat. However, every major scientific society worldwide has concluded that existing genetically modified crops are safe to eat (National Academies of Sciences, Engineering, and Medicine 2016). In more than four decades of research in many different organisms, there has never been a single case of harm to humans or ecosystems caused by the process of genetic engineering.

There is a clear and urgent need for sustainable agricultural practices and the technologies to support them. Ronald emphasized her view that cutting-edge techniques combined with ecological farming practices are central to addressing agriculture’s most pressing social, economic, and environmental questions.

Beyond laboratory work, Ronald suggested that scientists have another important role to play: to explain these technologies and the opportunities they offer, so that society understands their value in helping to feed the world and reducing harm to the environment.

## Amita Sehgal

### Mechanisms, functions, and regulation of sleep cycles

#### Findings from a Drosophila model:

Sleep is vital throughout the animal kingdom. Humans, for example, spend about one-third of our lives asleep, and insufficient or disordered sleep can have a huge impact on our health. Yet we lack a clear understanding of why sleep is necessary, the mechanisms behind it, and how it is regulated.

Amita Sehgal (University of Pennsylvania), studies circadian rhythms and other factors that drive rhythmic behaviors such as sleep. About 15 years ago, Sehgal developed a model for sleep using the fruit fly (*D. melanogaster*). In the team’s behavioral assay, individual flies in a glass tube are monitored constantly using an infrared beam; computers record this data and identify 5 min or more of immobility as time spent asleep. Using this technique, Sehgal and her colleagues have focused on deciphering the molecular and cellular underpinnings of how sleep is restricted to a certain time of day, the function of sleep, and the regulation of sleep onset and maintenance.

##### Mechanisms supporting sleep cycles:

Establishing regular rhythms requires a complicated circuit of neurons and peptides. Fly brains have six groups of so-called “clock” cells, and of these, a group known as small lateral ventral neurons (s-LNv) is critical for driving the rhythm of sleep and wake in flies. These clock cells extend to the dorsal part of the fly brain and produce a peptide called Pigment Dispersing Factor, which is also important for maintaining rhythms.

Building on this knowledge, Daniel Cavanaugh, a postdoctoral researcher working in Sehgal’s lab, was interested in examining which neurons in the fly brain other than the clock cells might play a role in determining rhythms. Cavanaugh’s results showed that the Pars Intercerebralis (PI), a group of neurons in the dorsal part of the fly brain, was required for maintaining rhythms (Cavanaugh *et al.* 2014). A polysynaptic circuit extends from central clock cells through other neurons in the brain to the PI, which enables daily timing.

In flies, the PI contains insulin-producing cells and also other peptidergic neurons, making it a neuroendocrine structure similar to the mammalian hypothalamus. Profiling the PI to determine other peptides that may be integral to driving sleep–wake cycles through communicating time of day signals, the team found that one such peptide is DH44, the *Drosophila* equivalent of corticotrophin-releasing factor (CRF), which is involved in the human stress response.

##### Sleep functions:

Little research had been done on why sleep is required, particularly in early life. Matthew Kayser, also a postdoctoral fellow in the Sehgal laboratory, has used the fruit fly model to study age-related differences in sleep. Kayser’s results supported the idea that young flies sleep more and fall asleep faster than their older counterparts (Kayser *et al.* 2014). The team also found that the mechanism behind this difference is dopamine. Dopamine is a critical chemical for many functions throughout the body, and one of its key effects is to produce arousal (in flies as well as mammals). Noting that older flies generally exhibit a higher level of dopamine in the brain, Kayser posits that this increased level of dopamine explains the greater wakefulness and reduced sleep seen in older *vs.* younger flies.

In addition to having lower levels of dopamine generally, the activity patterns of certain areas of the brain seem to promote sleep in younger flies. For example, the dorsal fanshaped body (dFSB), a region of the fly brain important for promoting sleep, is inhibited by dopaminergic inputs. In the case of young flies, the dopamine-transmitting neurons projecting into the dFSB are less active and thus promote greater amounts of sleep in the younger population compared to the older flies.

This age-related difference in sleep may be explained by the role sleep plays in development and behavior. In particular, sleep deprivation in young flies reduces mating when the flies are older. The research team found that experimentally stimulating dopamine neurons reduces dFSB activity and results in reduced sleep. This sleep loss in young flies reduces adult courtship, measured by the length of time male flies spend courting females, and decreases the percentage of males that follow through from courtship to copulation.

In an effort to study the specific circuits involved in this altered behavior, Sehgal examined the olfactory system, or sense of smell. Flies use smells and pheromones to orient to each other, giving olfaction a large role in the mating ritual. The processing of smell starts with sensory input to the antenna. The signal transfers through synapses in olfactory glomeruli in the antenna lobe before being processed by other structures.

After depriving both young and old flies of sleep by stimulating dopamine neurons, Sehgal’s group examined the size of the glomeruli. One particular glomerulus, VA1v, appeared to be sensitive to sleep loss in younger—but not older—flies. Unlike other areas, the VA1v glomerulus grows after the young flies have hatched. The strong effect of sleep deprivation on VA1v glomerulus size suggests that parts of the brain that grow posthatching are the areas that require the most sleep to properly develop, especially in the context of courtship circuitry.

##### Regulating sleep onset and maintenance:

Given sleep’s integral role in courtship behaviors, Sehgal sought to understand the mechanisms that regulate sleep. In this vein, she examined the neurotransmitters implicated in sleep. There is a great deal of overlap in the neurotransmitters that are involved in sleep in humans and flies, including dopamine, norepinephrine, serotonin, histamine, and γ-aminobutyric acid (GABA). In addition, sleep and insomnia-related neurological conditions in humans have been related to voltage-gated potassium channels and nicotinic acetylcholine receptor, both of which are also implicated in Sehgal’s *Drosophila* research.

Throughout their work, Sehgal and her colleagues have found unbiased approaches to studying animal behaviors, including forward genetics, to be extremely useful methods. *Drosophila* is a particularly well-suited model for forward genetics. In fact, the first circadian genes identified in *Drosophila* were found through this type of experimentation. Forward genetics in *Drosophila* is particularly effective for investigating sleep because sleep phenotypes are easily modified and in flies there is less redundancy and compensation, which enables researchers to isolate more robust mutant phenotypes. Using such unbiased approaches, Sehgal’s team was able to isolate a “sleepless” mutant fly, which sleeps 80% less than flies without the mutation. The mutant fly has been extremely useful in studying the effects of low sleep.

Sehgal’s team used these techniques to search for genes dedicated to sleep homeostasis. By conducting loss-of-function screens for mutants affecting daily sleep amount, Sehgal’s team found several genes that they determined were permissive for sleep, but not instructive, meaning they will allow but not induce sleep. Thus, they conducted an over-expression screen. Out of a screen of more than 12,000 genes, just one was implicated in inducing long sleep. Based on these findings, Sehgal believes single molecules that induce sleep will be rare.

Sehgal’s research using a *Dropsophila* model has shed light on many of the mysteries of sleep. Venturing into the neural circuitry behind the daily rhythms supporting sleep, her team was able to trace the connections among central clock cells and the PI and identify peptides such as DH44 that are required for rhythmic behavior. Seeking to examine the functions of sleep, the lab revealed the important role of sleep in developing the neural foundation for fruit fly courtship behaviors. Finally, using unbiased approaches including forward genetics to examine regulation of sleep onset and maintenance, the team found a gene, *nemuri*, that appears to induce sleep. Such studies advance understanding of the role of sleep across the animal kingdom and could perhaps improve our ability to prevent and treat sleep-related health disorders.

## Leonard Zon

### Translating zebrafish development to the clinic

#### Findings on blood stem cells and hematopoiesis:

Leonard Zon (HHMI/Harvard University/Boston Children’s Hospital) uses zebrafish as a model system to understand how new blood stem cells are formed and how they make new blood cells through hematopoiesis. Since hematopoiesis in zebrafish and humans is a very similar process, zebrafish are ideal for studying where new blood stem cells come from and how they create the billions of new blood cells a body needs daily. This work has led to some promising potential drug targets and treatments for leukemia, melanoma, and other diseases.

Blood stem cells can either self-renew or differentiate into all of the lineages of the peripheral blood cells. They are the only stem cells for which a quantitative assay is available, and are the only stem cells, besides skin stem cells, that are currently used therapeutically. By mapping the fate of new cells in zebrafish, Zon’s group has identified many of the steps involved in the formation and migration of new blood stem cells. They are also trying to better understand the biochemistry and dynamics involved in each of these steps.

##### Watching stem cell colonization:

The researchers are particularly interested in blood stem cell colonization because understanding this key but poorly understood process could provide insight into how to improve the success rates for bone marrow transplants. To watch stem cell colonization taking place, the researchers created a transgenic zebrafish with fluorescently marked stem cells, revealing the birth of the stem cells in the aorta and movement of the stem cells through the blood circulation before their landing in the caudal hematopoietic tissue. There, endothelial cells surround the stem cells and stem cell division takes place. Finally, one stem cell leaves the stem cell niche while the other stays (Tamplin *et al.* 2015).

**Figure 5:**
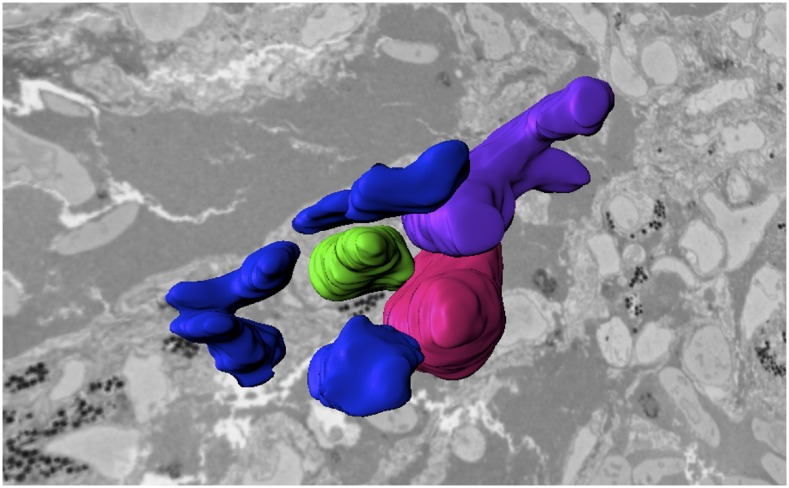
High-Resolution Electron Microscopy of Endogenous Hematopoietic Stem and Progenitor Cell (HSPC) in the Perivascular Niche. Lodged HSPC (green), surrounding EC (electron capture) nuclei (blue, numbered), and stromal cells (pink and purple). Reprinted from Tamplin *et al.* (2015), with permission from Elsevier.

To better define the stem cell niche, the researchers conducted correlative electron microscopy on the same transgenic zebrafish. This work produced the highest resolution picture available for a stem cell niche in a vertebrate species. It showed the stem cell surrounded by a pocket of endothelial cells, which may allow a higher concentration of growth factors or offer a protective space for the stem cell. Surprisingly, the stem cell was physically attached to a stromal cell (Figure 5). Using transgenic fish with labeled stromal and stem cells, Zon and colleagues found that the stromal cells help orient the plane of stem cell division, with most divisions occurring perpendicular to the stromal cell plane. Once the stem cell emerges through the endothelial wall into its niche, a macrophage begins interacting with the stem cell. They also saw that a second macrophage is responsible for escorting the stem cell to the stromal cell.

To determine how many stem cells are born in the aorta, “brainbow” fish were mated to a blood-specific CreER^t2^ line and Tamoxifen added at multiple times during development to produce multiply-labeled embryonic blood. By adulthood, all the embryonic blood is replaced with stem cell-derived blood. Although Zon’s group thought that counting the number of colors would let them back-calculate the number of stem cells born in the aorta, they realized they would need controls showing what each color looks like alone. Thus, they decided to use a ubiquitous Cre label to make every sperm cell a different color, yielding zebrafish embryos that are one of many colors. Fluorescence-activated cell sorting of the fish embryos revealed that 21 stem cells are born in the dorsal aorta, which suggests that a pool of stem cells is amplified in each stem cell niche.

The researchers are also using these multi-color zebrafish to study a disorder known as clonal hematopoiesis of indeterminate potential (CHIP), which predisposes people to leukemia. With increasing age, the percentage of people with mutations in genes that cause clonal expansion increases, but scientists do not know why this causes a predisposition to leukemia. The researchers injected CHIP disease-causing genes or knocked out these genes to see if they could make the multiclonal (multicolor) fish monoclonal (one color). This work revealed that mutations in the *Asxl-1* gene are strongly tied to clonal expansion, enabling a search for molecules that might suppress the clonal expansion. They also found that different combinations of mutations result in different syndromes.

##### Improving leukemia treatment:

Bone marrow transplants are used today as a treatment for leukemia, but about 25% of patients die from the procedure. The patient first receives chemotherapy to erase the leukemia and then receives blood stem cells, typically from a relative, to rebuild the immune system. The transplanted stem cells make their way to the bone marrow where engraftment takes place.

The researchers used zebrafish to find molecules that would increase the number of blood stem cells that would go on to produce blood cells after a transplant. One such molecule was also demonstrated to be successful in mice and is now in phase II clinical trials. The clinical trials have been promising so far. In addition to showing an increase in the number of stem cells, the patients receiving the treatment also experienced lower rates of graft *vs.* host disease, as well as less viral reactivation after the transplant (Cutler *et al.* 2013). Meanwhile, Zon’s team is continuing to use zebrafish to study the nuances of how bone marrow engraftment occurs to further improve treatments. Some of the zebrafish experiments are revealing that inflammatory lipids are important in how blood stem cells traffic (Li *et al.* 2015).

##### Finding treatments for other diseases:

Zon and colleagues are also using zebrafish to look for new treatments for rare ribosomopathies, which underlie several rare genetic diseases. Ribosomopathies result from defects in ribosome biogenesis that activate P53 and lead to cell death. They used a zebrafish line with a *rps29* mutation, which causes a hemoglobin defect, to screen for potentially therapeutic molecules. Patients with this ribosome mutation have a disease called Diamond-Blackfan anemia, in which the bone marrow fails to make red blood cells. Very few therapy options exist for these patients except for transplantation. The zebrafish screen showed that the calmodulin inhibitor TFP, an antipsychotic in current use, completely reversed the effects of this mutation in zebrafish. Experiments with the drug in mouse and *in vitro* human models of Diamond-Blackfan anemia were also successful, and a clinical trial is slated to begin in 2017.

Zon is also working to understanding melanoma and to find potential treatments for this skin cancer using zebrafish. By modeling melanoma in the zebrafish, they have found that a gene called *crestin* marks all the migrating neural crest cells in zebrafish embryos. In adult fish this gene is typically repressed, but when melanoma is present, *crestin* is expressed. GFP-tagged crestin labeled melanoma cells and allowed their detection before clinical melanoma was obvious in the fish. This is likely the first time that cancer has been observed in a vertebrate at the single-cell stage. *Crestin* activated in human melanoma was similar to that observed in the zebrafish preclinical patches. Crestin reactivation could provide a way to identify precancerous moles or offer a therapeutic target for repression of genes involved in melanoma formation and progression. Further studies showed that SATB2 overexpression leads to cranial neural crest development and cell migration, initiating cell migration programs and ultimately metastasis. Chemical screening revealed that the drug leflunomide can block neural crest development. This drug is now in clinical trials.

To see if zebrafish could be used as a clinical translational model, Zon’s team developed an oral gavage procedure to direct drugs into the stomach of the zebrafish. This method offers an inexpensive way to test drugs, especially when compared to xenograft studies, which are valuable but expensive. Doing fish studies before moving to xenograft studies could be a useful and money-saving combination approach.

## Honors and Awards

### GSA Awards

#### Thomas Hunt Morgan Medal

Nancy Kleckner (Harvard University)

#### Genetics Society of America Medal

Detlef Weigel (Max Plank Institute for Developmental Biology)

#### George W. Beadle Award

Susan E. Celniker (Lawrence Berkeley National Laboratory)

#### Elizabeth W. Jones Award for Excellence in Education

William Wood (University of Colorado)

#### The Novitski Prize

Leonid Kruglyak (HHMI and University of California, Los Angeles)

#### The Rosalind Franklin Young Investigator Award

Maria Barna (Stanford University)

Carolyn McBride (Princeton University)

### GSA Undergraduate Travel Awards

Cynthia Becker (Ithaca College).

Diana Cardero (Florida International University).

Brian Choi (Indiana University).

Logan Condon (University of Washington).

Max Haase (University of Wisconsin–Madison).

Mathieu Henault (Laval University).

Alex Lederer (University of Pittsburgh).

Xiaoxue Lin (College of William and Mary).

Maegan Neilson (College of the Holy Cross).

Erin Ritchie (University of Utah).

Carolyn Turcotte (Marist College).

### C. elegans Development, Cell Biology, and Gene Expression Meeting

#### Poster Awards:

##### Graduate students:

1st Place: Kimberly Gauthier (MUHC Research Institute, McGill University).

2nd Place: Cristina Matthewman (University of Miami Miller College of Medicine).

3rd Place: Amel Alqadah (University of Illinois at Chicago).

##### Undergraduate students:

1st Place: James Brandt (Lewis & Clark College).

2nd Place*:* Maegan Neilson and Lauren Riley (College of the Holy Cross).

### Ciliate Molecular Biology Conference

#### Poster Awards:

##### Graduate student:

Miguel Gonzales (Texas A&M University).

##### Undergraduate student:

Evan Wilson (Missouri State University).

### 57th Annual Drosophila Research Conference

#### Larry Sandler Memorial Lecture:

Alejandra Figueroa-Clarevega (University of California, Berkeley).

#### Drosophila Image Award:

##### Winners:

Raghav Chhetri (Janelia Research Campus, HHMI).

Tanya Wolff (Janelia Research Campus, HHMI).

##### Runners-up:

Jonathan Enriquez (Columbia University).

Justin Bosch (Harvard University).

#### Poster Awards:

##### Graduate students:

1st Place: Daniel Kelpsch (University of Iowa).

2nd Place: Afsoon Saadin (University of Maryland Baltimore County).

3rd Place: Sumaira Zamurrad (Albert Einstein College of Medicine).

##### Undergraduate students:

1st Place: Niahz Wince (Pennsylvania State University, Berks College).

2nd Place: Phuong Nguyen (Drexel University).

#### Victoria Finney Undergraduate Travel Awards:

Andrew Blake (Delaware State University).

Taylor Hinnant (East Carolina University).

Diana Luong (Loyola University Chicago).

Katherine Nichols (Muhlenberg College).

Abigail O’Conner (University of Arizona).

Nilang Shah (Emory University).

Jarrod Shilts (Vanderbilt University).

Samantha St. Clair (Indiana University).

### 2016 Mouse Genetics Conference

#### IMGS Verne Chapman Young Investigator Prize:

Jacob Moskowitz (University of Missouri).

#### IMGS Mary Lyon Award:

Krista Geister (Seattle Children’s Research Institute).

#### IMGS Oral Presentation Awards:

Elijah Edmondson (Colorado State University)

Nitha Kartha (Cornell University)

#### GSA Poster Awards:

##### Graduate students:

1st Place: Meng Zhang (Yale University).

2nd Place: Sarah Bay (Emory University).

##### Undergraduate students:

Andreea Radulescu (University of Surrey, UK).

#### IMGS Poster Awards:

Mark Webster (Western Michigan University)

William Barrington (Texas A&M University)

Nirav Chhabra (HelmholtzZentrum Munchen, Germany)

Sehoon Keum (Institute for Basic Science, Korea)

Lucas Laudermilk (University of North Carolina, Chapell Hill)

Sarah Lewis (University of Wisconsin, Madison)

Stephen Wellard (Johns Hopkins Bloomberg School of Public Health)

Sofia Ivanatsiv (University of Toronto)

Shigeru Makino (RIKEN BioResource Center, Japan)

Samantha Fletcher (Texas A&M University)

### Population, Evolutionary, and Quantitative Genetics

#### James F. Crow Early Career Researcher Award:

Sarah E. Sander (Cornell University).

#### Poster Awards:

##### Graduate students:

1st Place: Thom Nelson (University of Oregon).

2nd Place: Michelle Parmenter (University of Wisconsin–Madison).

3rd Place: April Peterson (University of Wisconsin–Madison).

##### Undergraduate student:

Mathieu Henault (IBIS Université Laval, Canada).

### Yeast Genetics Meeting

#### Yeast Genetics Meeting Lifetime Achievement Award:

James Broach (Penn State University).

#### Ira Herskowitz Award:

Lars Steinmetz (Stanford University/EMBL).

#### Winge-Lindegren Address:

Rodney Rothstein (Columbia University).

#### Lee Hartwell Lecture:

Susan Gasser, (Friedrich Miescher Institute).

#### Poster Awards:

##### Graduate students:

1st Place: Dara Lo (University of Toronto, Canada).

2nd Place (tied): Jon Laurent (University of Texas at Austin)and Yu-San Yang (University of Texas Southwestern Medical Center).

##### Undergraduate student:

Alex Lederer (University of Pittsburgh).

### 12th International Conference on Zebrafish Development and Genetics

#### Chi-Bin Chien Award:

Adam Miller (University of Oregon).

#### George Streisinger Award:

Chuck Kimmel (University of Oregon).

#### International Zebrafish Society Poster Awards

##### Postdoctoral researchers

Chi-Kuo Hu (Stanford University)

Kazunori Okada (Okazaki Institute for Integrative Biology/National Institute for Basic Biology)

Ivan Cruz (University of Washington)

##### Graduate students

Molly Matty (Duke University)

Tyson Fuller (University of Iowa)

Emilia Asante (University of Missouri)

##### Undergraduate students

Mike Waltman (NIH/NIDCD)

Logan Condon (University of Washington)

#### EuFishBioMed Poster Award

Zhen Jiang (University of Sheffield)

#### Travel Awards

Mitchell D'Rozario (Washington University School of Medicine)

Yaniv M Elkouby (University of Pennsylvania)

Meagan Grant (Princeton University)

Michael R.M. Harrison (Children's Hospital Los Angeles)

Sundas Ijaz (Yale University)

Junsu Kang (Duke University)

Daniel A Lee (California Institute of Technology)

Zairan Liu (University California, San Francisco)

Braedan M McCluskey (University of Oregon)

Jose L. Pelliccia (Princeton University)

Tetiana Petrachkova (Western Michigan University)

Dorien Schepers (University of Antwerp and Antwerp University Hospital)

Jinelle Wint (Stony Brook University)

## References

[bib1] Andersen E. C. Gerke J. P. Shapiro J. A. Crissman J. R. Ghosh R., 2012 Chromosome-scale selective sweeps shape *Caenorhabditis elegans* genomic diversity. Nat. Genet. 44(3): 285–290. 22286215 10.1038/ng.1050 PMC3365839

[bib2] Auton A. Fledel-Alon A. Pfeifer S. Venn O. Ségurel L., 2012 A fine-scale chimpanzee genetic map from population sequencing. Science 336(6078): 193–198. 22422862 10.1126/science.1216872 PMC3532813

[bib3] Boeke J. D. Church G. Hessel A. Kelley N. J. Arkin A., 2016 Genome engineering. The genome project-write. Science 353(6295): 126–127. 27256881 10.1126/science.aaf6850

[bib4] Bourne P. E. Lorsch J. R. Green E. D., 2015 Perspective: sustaining the big-data ecosystem. Nature 527(7576): S16–S17. 26536219 10.1038/527S16a

[bib5] Cavanaugh D. J. Geratowski J. D. Wooltorton J. R. A. Spaethling J. M. Hector C. E., 2014 Identification of a circadian output circuit for rest: activity rhythms in *Drosophila*. Cell 157(3): 689–701. 24766812 10.1016/j.cell.2014.02.024 PMC4003459

[bib6] Chan Y. F. Marks M. E. Jones F. C. Villarreal G. Jr. Shapiro M. D., 2010 Adaptive evolution of pelvic reduction in sticklebacks by recurrent deletion of a Pitx1 enhancer. Science 327(5963): 302–305. 20007865 10.1126/science.1182213 PMC3109066

[bib7] Chen X. Bracht J. R. Goldman A. D. Dolzhenko E. Clay D. M., 2014 The architecture of a scrambled genome reveals massive levels of genomic rearrangement during development. Cell 158(5): 1187–1198. 25171416 10.1016/j.cell.2014.07.034 PMC4199391

[bib8] Cheung B. H. Arellano-Carbajal F. Rybicki I. de Bono M., 2004 Soluble guanylate cyclases act in neurons exposed to the body fluid to promote *C. elegans* aggregation behavior. Curr. Biol. 14(12): 1105–1111. 15203005 10.1016/j.cub.2004.06.027

[bib9] Collins F. S. Anderson J. M. Austin C. P. Battey J. F. Birnbaum L. S., 2016 Basic science: bedrock of progress. Science 351(6280): 1405. 10.1126/science.351.6280.1405-a PMC5101936 27013720

[bib10] Coop G. Wen X. Ober C. Pritchard J. K. Przeworski M., 2008 High-resolution mapping of crossovers reveals extensive variation in fine-scale recombination patterns among humans. Science 319(5868): 1395–1398. 18239090 10.1126/science.1151851

[bib11] Crosland M. W. Crozier R. H., 1986 *Myrmecia pilosula*, an ant with only one pair of chromosomes. Science 231(4743): 1278. 17839565 10.1126/science.231.4743.1278

[bib12] Cutler C. Multani P. Robbins D. Kim H. T. Le T., 2013 Prostaglandin-modulated umbilical cord blood hematopoietic stem cell transplantation. Blood 122(17): 3074–3081. 23996087 10.1182/blood-2013-05-503177 PMC3811179

[bib13] de Bono M. Bargmann C. I., 1998 Natural variation in a neuropeptide Y receptor homolog modifies social behavior and food response in *C. elegans*. Cell 94(5): 679–689. 9741632 10.1016/s0092-8674(00)81609-8

[bib14] Doak T. G. Cavalcanti A. R. O. Stover N. A. Dunn D. M. Weiss R., 2003 Sequencing the *Oxytricha trifallax* macronuclear genome: a pilot project. Trends Genet. 19(11): 603–607. 14585610 10.1016/j.tig.2003.09.013

[bib15] Fang W. Wang X. Bracht J. R. Nowacki M. Landweber L. F., 2012 Piwi-interacting RNAs protect DNA against loss during *Oxytricha* genome rearrangement. Cell 151(6): 1243–1255. 23217708 10.1016/j.cell.2012.10.045 PMC3678556

[bib16] Gardner M. R. Kattenhorn L. M. Kondur H. R. von Schaewen M. Dorfman T., 2015 AAV-expressed eCD4-Ig provides durable protection from multiple SHIV challenges. Nature 519(7541): 87–91. 25707797 10.1038/nature14264 PMC4352131

[bib17] Gray J. M. Karow D. S. Lu H. Chang A. J. Chang J. S., 2004 Oxygen sensation and social feeding mediated by a *C. elegans* guanylate cyclase homologue. Nature 430(6997): 317–322. 15220933 10.1038/nature02714

[bib18] Grimes D. T. Boswell C. W. Morante N. F. Henkelman R. M. Burdine R. D., 2016 Zebrafish models of idiopathic scoliosis link cerebrospinal fluid flow defects to spine curvature. Science 352(6291): 1341–1344. 27284198 10.1126/science.aaf6419 PMC5574193

[bib19] Guenther C. A. Tasic B. Luo L. Bedell M.A. Kingsley D. M., 2014 A molecular basis for classic blond hair color in Europeans. Nat. Genet. 46: 748–752. 24880339 10.1038/ng.2991 PMC4704868

[bib20] Han S. Lee S. Bahar O. Schwessinger B. Robinson M. R., 2012 Tyrosine sulfation in a Gram-negative bacterium. Nat. Commun. 3: 1153. 23093190 10.1038/ncomms2157 PMC4305400

[bib21] Jinek M. Chylinski K. Fonfara I., 2012 A programmable dual-RNA-guided DNA endonuclease in adaptive bacterial immunity. Science 337(6096): 816–821. 22745249 10.1126/science.1225829 PMC6286148

[bib22] Jones F. C. Grabherr M. G. Chan Y. F. Russell P. Mauceli E., 2012 The genomic basis of adaptive evolution in threespine sticklebacks. Nature 484(7392): 55–61. 22481358 10.1038/nature10944 PMC3322419

[bib23] Kayser M. S. Yue Z. Sehgal A., 2014 A critical period of sleep for development of courtship circuitry and behavior in *Drosophila*. Science 344(6181): 269–274. 24744368 10.1126/science.1250553 PMC4479292

[bib24] Knight S. C. Xie L. Deng W. Guglielmi B. Witkowsky L. B., 2015 Dynamics of CRISPR-Cas9 genome interrogation in living cells. Science 350(6262): 823–826. 26564855 10.1126/science.aac6572

[bib25] Lauer, M., 2016 A Look at Trends in NIH’s Model Organism Research Support. Available at: https://nexus.od.nih.gov/all/2016/07/14/a-look-at-trends-in-nihs-model-organism-research-support/. Accessed: September 30, 2016.

[bib26] Li P. Lahvic J. L. Binder V. Pugach E. K. Riley E. B., 2015 Epoxyeicosatrienoic acids enhance embryonic haematopoiesis and adult marrow engraftment. Nature 523: 468–471. 26201599 10.1038/nature14569 PMC4754787

[bib27] Maître J.-L. Turlier H. Illukkumbura R. Eismann B. Niwayama R., 2016 Asymmetric division of contractile domains couples cell positioning and fate specification. Nature 536: 344–348. 27487217 10.1038/nature18958 PMC4998956

[bib28] Miller C. T. Beleza S. Pollen A. A. Schluter D. Kittles R. A., 2007 cis-Regulatory changes in Kit ligand expression and parallel evolution of pigmentation in sticklebacks and humans. Cell 131(6): 1179–1189. 18083106 10.1016/j.cell.2007.10.055 PMC2900316

[bib29] Mitchell L. A. Chuang J. Agmon N. Khunsriraksakul C. Phillips N. A., 2015 Versatile genetic assembly system (VEGAS) to assemble pathways for expression in *S. cerevisiae*. Nucleic Acids Res. 43(13): 6620–6630. 25956652 10.1093/nar/gkv466 PMC4513848

[bib30] Möllenbeck M. Zhou Y. Cavalcanti A. R. O. Jönsson F. Higgins B. P., 2008 The pathway to detangle a scrambled gene. PLoS One 3(6): e2330. 18523559 10.1371/journal.pone.0002330 PMC2394655

[bib31] National Academies of Sciences, Engineering, and Medicine, 2016 *Genetically Engineered Crops: Experiences and Prospects*. The National Academies Press, Washington, D.C. 28230933

[bib32] Neurohr G. Naegeli A. Titos I. Theler D. Greber B., 2011 A midzone-based ruler adjusts chromosome compaction to anaphase spindle length. Science 332(6028): 465–468. 21393511 10.1126/science.1201578

[bib33] NIH Working Group on Data and Informatics, 2013 NIH Request For Information: Management, Integration, And Analysis of Large Biomedical Datasets. Bethesda, MD: National Institutes of Health. Available at: http://acd.od.nih.gov/DIWG_RFI_FinalReport.pdf. Accessed: September 30, 2016.

[bib34] O’Brown N. M. Summers B. R. Jones F. C. Brady S. D. Kingsley D. M., 2015 A recurrent regulatory change underlying altered expression and Wnt response of the stickleback armor plates gene EDA. eLife 4: e05290. 25629660 10.7554/eLife.05290 PMC4384742

[bib35] Pruitt R. N. Schwessinger B. Joe A. Thomas N. Liu F., 2015 The rice immune receptor XA21 recognizes a tyrosine-sulfated protein from a Gram-negative bacterium. Sci. Adv. 1(6): e1500245. 26601222 10.1126/sciadv.1500245 PMC4646787

[bib36] Ptak S. E. Roeder A. D. Stephens M. Gilad Y. Pääbo S., 2004 Absence of the TAP2 human recombination hotspot in chimpanzees. PLoS Biol. 2(6): e155. 15208713 10.1371/journal.pbio.0020155 PMC423135

[bib47] Ptak S. E. Hinds D. A. Koehler K. Nickel B. Patil N., 2005 Fine-scale recombination patterns differ between chimpanzees and humans. Nat. Genet. 37: 429–434. 15723063 10.1038/ng1529

[bib37] Reddy K. C. Andersen E. C. Kruglyak L. Kim D. H., 2009 A polymorphism in npr-1 is a behavioral determinant of pathogen susceptibility in *C. elegans*. Science 323(5912): 382–384. 19150845 10.1126/science.1166527 PMC2748219

[bib38] Reischauer S. Stone O. A. Villasenor A. Chi N. Jin S. W., 2016 Cloche is a bHLH-PAS transcription factor that drives haemato-vascular specification. Nature 535(7611): 294–298. 27411634 10.1038/nature18614

[bib39] Robertson H. M. Thomas J. H., 2006 The putative chemoreceptor families of *C. elegans*. WormBook 6: 1–12. 10.1895/wormbook.1.66.1 PMC4781013 18050473

[bib40] Rogers C. Reale V. Kim K. Chatwin H. Li C., 2003 Inhibition of *Caenorhabditis elegans* social feeding by FMRFamide-related peptide activation of NPR-1. Nat. Neurosci. 6(11): 1178–1185. 14555955 10.1038/nn1140

[bib41] Shen Y. Stracquadanio G. Wang Y. Yang K. Mitchell L. A., 2016 SCRaMbLE generates designed combinatorial stochastic diversity in synthetic chromosomes. Genome Res. 26(1): 36–49. 26566658 10.1101/gr.193433.115 PMC4691749

[bib42] Song W. Y. Wang G. L. Chen L. L. Kim H. S. Pi L. Y., 1995 A receptor kinase-like protein encoded by the rice disease resistance gene, Xa21. Science 270(5243): 1804–1806. 8525370 10.1126/science.270.5243.1804

[bib43] Sulem P. Gudbjartsson D. F. Stacey S. N. Helgason A. Rafnar T., 2007 Genetic determinants of hair, eye and skin pigmentation in Europeans. Nat. Genet. 39(12): 1443–1452. 17952075 10.1038/ng.2007.13

[bib44] Swart E. C. Bracht J. R. Magrini V. Minx P. Chen X., 2013 The *Oxytricha trifallax* macronuclear genome: a complex eukaryotic genome with 16,000 tiny chromosomes. PLoS Biol. 11(1): e1001473. 23382650 10.1371/journal.pbio.1001473 PMC3558436

[bib45] Tamplin O. J. Durand E. M. Carr L. A. Childs S. J. Hagedorn E. J., 2015 Hematopoietic stem cell arrival triggers dynamic remodeling of the perivascular niche. Cell 160(1–2): 241–252. 25594182 10.1016/j.cell.2014.12.032 PMC4346256

[bib46] Wangler M. F. Yamamoto S. Bellen H. J., 2015 Fruit flies in biomedical research. Genetics 199(3): 639–653. 25624315 10.1534/genetics.114.171785 PMC4349060

